# Shape Sensing of Post-Buckling Shear Panels Using Distributed Optical Fiber Sensors and Ko’s Displacement Theory: Comparison with Digital Image Correlation †

**DOI:** 10.3390/s26206611

**Published:** 2026-10-19

**Authors:** Pietro Aceti, Sebastian Stammel, Giuseppe Sala, Chiara Bisagni

**Affiliations:** Department of Aerospace Science and Technology, Politecnico di Milano, Via La Masa 34, 20156 Milano, Italy; sebastian.stammel@polimi.it (S.S.); giuseppe.sala@polimi.it (G.S.); chiara.bisagni@polimi.it (C.B.)

**Keywords:** shape reconstruction, digital image correlation, distributed optical fiber sensors, post-buckling, Wagner beam, Ko’s displacement theory

## Abstract

This paper presents a comparison of out-of-plane displacement measurement techniques for shear-loaded panels, using both a digital image correlation system and a distributed optical fiber sensor system. The digital image correlation system directly determines displacement and strain, while the distributed optical fiber sensor uses Ko’s displacement theory to compute displacement based on measured strain. A Wagner beam test rig is used to perform experimental investigations. The digital image correlation system determines the out-of-plane displacement of the panels, although it is currently suitable only for laboratory applications. Conversely, the distributed optical fiber sensor system offers the significant advantage of being embedded within structures, with potential for future continuous in-flight structural health monitoring. The displacement estimation by the distributed optical system is in agreement with the digital image correlation measurement, excluding the boundary area of the panels, where an extension of Ko’s displacement theory for this specific testing case is required.

## 1. Introduction

Structural displacement monitoring plays a crucial role in the design, operation, and maintenance of future aerospace structures, given the increasing use of lightweight structures, flexible wings, composite materials, and morphing structural elements. Strain monitoring improves the ability to detect damage in real time and, thereby improving both maintenance services and structural performance [1,2]. Moreover, shape detection can enable real-time evaluation of structural displacements and the implementation of a control system for morphing structures [3,4].

There are multiple methods to measure structural displacement. Digital image correlation (DIC) systems measure structural displacement directly via optical imaging [5,6]. This system is effective for laboratory activities and test flights; however, it is impractical for operational application in weight-conscious lightweight aerospace flying structures. Instead, recent research has shown that distributed optical fiber sensors (DOFS) are an excellent tool for strain measurement in aerospace structures. Such sensors enable continuous real-time strain monitoring, and do not introduce significant weight; in addition, the optical fibers can be bonded to lightweight metal components or embedded in composite materials during manufacturing, becoming an integral part of the structure. The technological aspects of this integration, including bonding and embedding techniques, compatibility with the host material, and thermal–mechanical signal decoupling, have been investigated for aeronautical applications [7]. This integration enables continuous monitoring of the component from the production phase through its entire operational life, using the measured strain to compute the structural displacement [8,9].

Different methods can be found in the literature to compute the shape displacement based on the strain distribution, among them:

- Ko’s displacement theory [10,11], which integrates the experimentally measured strain to compute the displacement.
- The modal method [12,13], which describes the displacement through a set of basis functions in which the set is selected using the measured strain distribution.
- The inverse finite element method [14,15], which works by applying a finite-element characterization and the variational principle.

The modal method requires prior derivation of the basis functions for the test structure, while the inverse finite element method is computationally intensive. The extensive effort needed to set up these methods contrasts with the simplicity of by Ko’s displacement theory, which uses closed-form equations derived from the classical Bernoulli–Euler beam theory to reconstruct the structural displacement of a wing structure based on measured strains at discrete positions [11,16,17,18]. Double integration provides the displacement at the same discrete locations; the displacements at those locations are only in the out-of-plane direction, not the in-plane direction, and are referred to as shifted displacement transfer functions. The closed-form equations require only knowledge of the structural geometric features. In the literature, this method has been applied to a doubly-tapered wing of an unmanned vehicle, where the results for the out-of-plane displacement and twist angle showed high precision compared to a finite-element model [19]. A further step conducted experimental verification [20] with a ground load test of a full-scale wing from the Global Observer Unmanned Aerial Vehicle. This method has also been applied to compute the chordwise and spanwise out-of-plane displacements of a wing using strain measurements from a distributed fiber optic system [21]. The transfer functions used in Ko’s displacement theory have been extended to include both out-of-plane and in-plane displacements in order to predict the structural shape under large geometrically nonlinear displacements, where they are referred to as curved displacement transfer functions [22,23]. Validation of the curved displacement transfer functions was performed with high accuracy for the sample case of a tapered cantilever tubular beam [23].

Recent research [24] has enabled full-field displacement estimation through a two-step process using a modal transformation. In the first step, the displacement along the strain-sensing lines is computed using Ko’s traditional displacement theory; a second step introduces the SEREP modal transformation [25] to extend shape sensing to the entire structure. An expansion to include geometrically nonlinear shape sensing was proposed and validated in [26] using the two-step approach, with the X-59 stabilator as a sample case, and achieved a prediction error of less than 0.3% for the wing-tip displacement.

As an alternative to the two-step approach, a full-field displacement estimation without considering modal transformation has been demonstrated as well [27,28]. The plate is divided into multiple narrow stripes, with each stripe considered separately for Ko’s displacement theory before being reassembled. This approach was validated for a plate under bending, finding good agreement with optical measurements for large displacements. Ko’s displacement theory was selected for the present study because it enables direct strain-to-displacement reconstruction along the sensing paths without requiring a predefined modal basis or a finite-element model.

In this paper, two methods are used to measure the out-of-plane displacement of shear-loaded panels in a Wagner beam undergoing post-buckling out-of-plane deformation: DIC measurement and DOFS displacement computation. The DOFS displacement is evaluated by measuring the strain along multiple lines on the panel and applying Ko’s displacement theory to each line. This comparison provides a preliminary basis for evaluating the compatibility of optical fibers for the practical application of measuring structural shape in aerospace structures. Structural shape measurements are essential for monitoring and controlling shape-adaptive aerospace structures that use elastic instabilities to switch between different structural shapes [29,30]. This paper extends the preliminary study presented at IWSHM 2025 [31] with an expanded theoretical framework and experimental analysis, including the influence of boundary effects and membrane strains on displacement reconstruction. In Section 2, we explain Ko’s displacement theory; following this, the experimental setup of the Wagner beam, the DIC and DOFS measurement, and the experimental procedure are explained in Section 3; finally, Section 4 presents and discusses the results.

## 2. Ko’s Displacement Theory

This section introduces the theoretical framework used for strain-based reconstruction of out-of-plane displacements from distributed strain measurements. The methodology is based on Ko’s displacement theory [11,19], which relates measured bending strains to structural curvature and displacement through classical beam theory. The formulation provides an efficient approach for reconstructing structural deformation using strain measurements and geometric information, making it particularly suitable for lightweight aerospace structures equipped with DOFS.

The derivation is first introduced for a cantilever beam subjected to bending in Section 2.1 and Section 2.2 and subsequently extended to plate structures in Section 2.3. Finally, the applicability of the method to post-buckled shear-loaded plates is discussed in Section 2.4.

### 2.1. Beam-Based Displacement Reconstruction

Ko’s displacement theory is derived from the classical Euler–Bernoulli beam formulation under the assumptions of isotropic linearly elastic material behavior, small strains, negligible transverse shear deformation, and plane sections that remain planar during deformation [32,33]. For a beam subjected to bending, the governing differential equation for the out-of-plane displacement $u_{z}\left(x\right)$ is

(1) $$\frac{d^{2}u_{z}\left(x\right)}{d^{2}x}=\frac{M(x)}{EI},$$

where M(x) denotes the bending moment, *E* the Young’s modulus, and *I* the second moment of the area. The bending stress σ(x) at the outer surface of the beam is given by

(2) $$\sigma \left(x\right)=\frac{M(x)h}{I},$$

where *h* represents the distance between the neutral axis and the outer beam surface. Using Hooke’s law, the corresponding bending strain ε(x) becomes

(3) $$\varepsilon \left(x\right)=\frac{\sigma (x)}{E}=\frac{M(x)h}{EI}.$$

**Figure 1:**
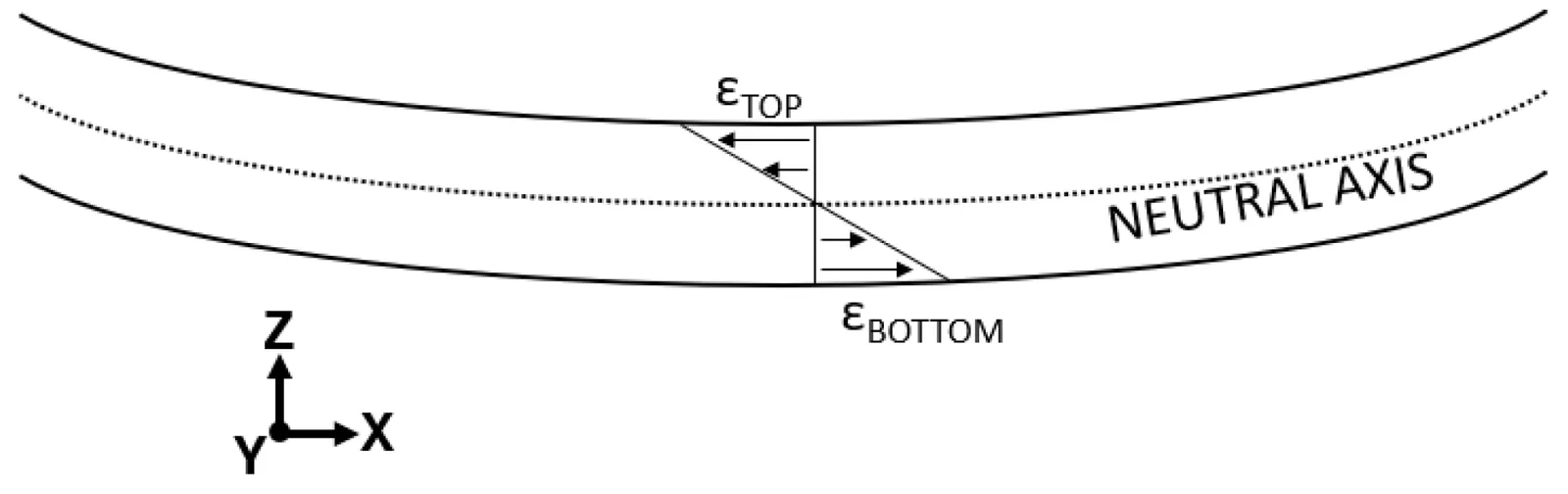
Definition of the bending strain $\varepsilon_{Bending}$ on a beam.

The strain input required for Ko’s displacement theory corresponds to the bending strain contribution; however, experimentally measured surface strains contain both bending and membrane strain components. The bending strain can be isolated by averaging the strain measurements obtained from the upper and lower surfaces of the structure (as shown in Figure 1):

(4) $$\varepsilon_{bending}=\varepsilon_{total}-\varepsilon_{axial}=\frac{\varepsilon_{bottom}-\varepsilon_{top}}{2}.\;$$

Substituting Equation (3) into Equation (1) yields the curvature relation:

(5) $$\frac{d^{2}u_{z}\left(x\right)}{d^{2}x}=\frac{\varepsilon (x)}{h}=\kappa$$

where κ(x) denotes the beam curvature. This formulation depends only on the measured strain and the beam geometry, without requiring explicit knowledge of the flexural rigidity EI.

The beam slope θ(x) between two locations *a* and *x* is obtained by integration of the curvature:

(6) $$tan\theta \left(x\right)=\int_{a}^{x}\frac{\varepsilon (x)}{h}dx+tan\theta \left(a\right).$$

**Figure 2:**
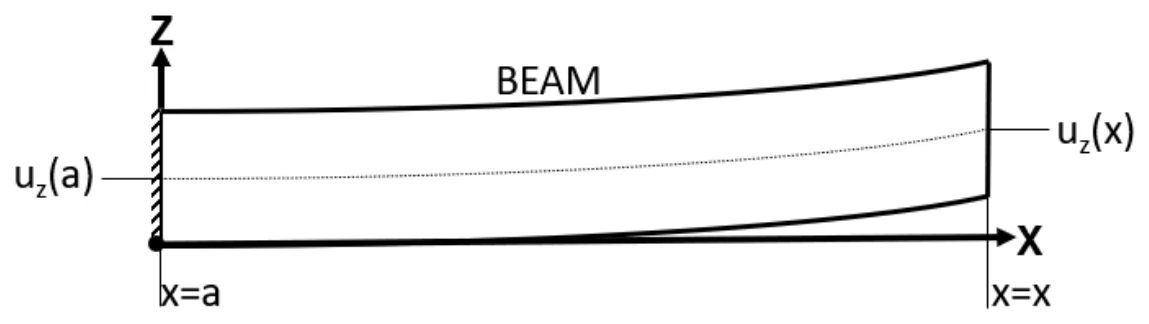
Out-of-plane displacement $u_{z}$ of a beam.

The corresponding out-of-plane displacement $u_{z}\left(x\right)$ is determined through a second integration (shown in Figure 2):

(7) $$u_{z}\left(x\right)=u_{z}\left(a\right)+\left(x-a\right)\tan\theta \left(a\right)+\int_{a}^{x}\int_{a}^{x}\frac{\varepsilon (x)}{h}d^{2}x.\;$$

This formulation is referred to as the shifted displacement formulation, since the displacement is reconstructed only in the out-of-plane direction while neglecting in-plane motion along the beam axis [23].

### 2.2. Curved Displacement Formulation

For structures undergoing large deformation, the assumption of purely vertical displacement becomes insufficient. In addition to the out-of-plane displacement, material points also experience in-plane motion during deformation [22,23]. To account for this behavior, the shifted displacement formulation is extended using a curvilinear coordinate system $s-{\hat{u}}_{z}$.

**Figure 3:**
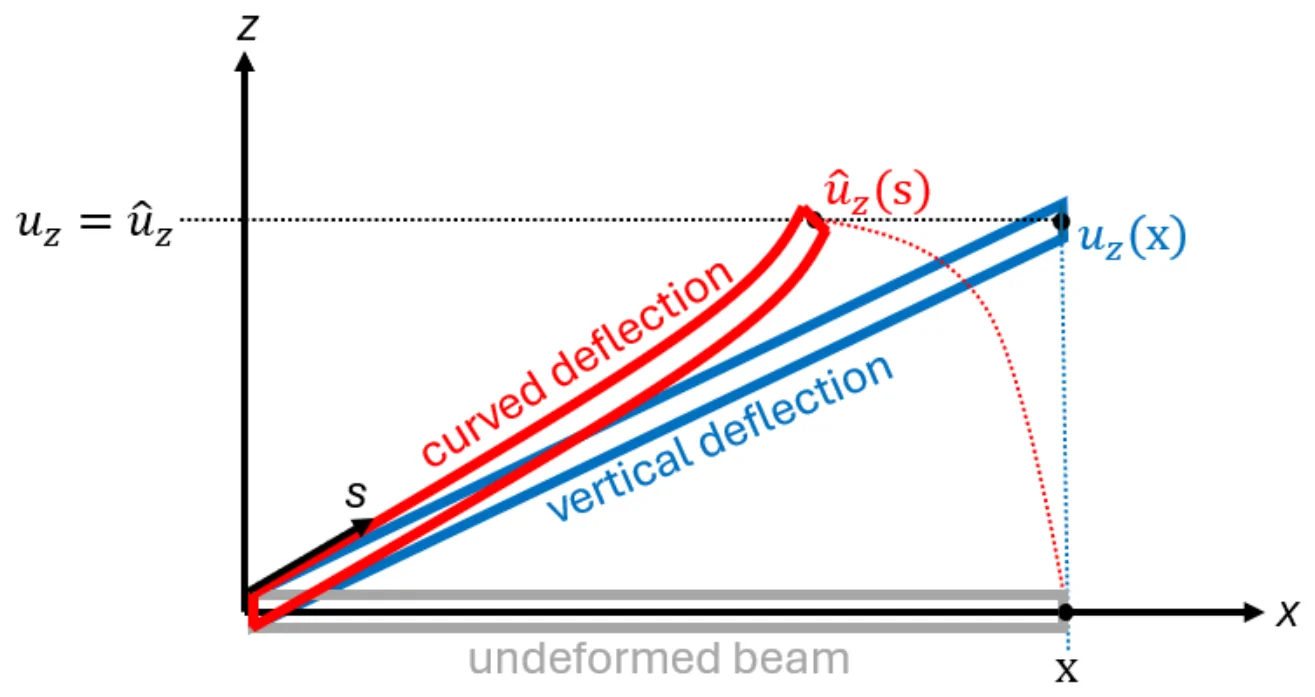
Out-of-plane displacement of an elastic beam with curved ${\hat{u}}_{z}$ and shifted $u_{z}$ displacement of the beam.

Figure 3 illustrates the difference between the shifted displacement formulation $u_{z}\left(x\right)$ and the curved displacement formulation ${\hat{u}}_{z}\left(s\right)$, where *s* denotes the curvilinear coordinate along the deformed neutral axis.

The curvature–displacement relation in the curvilinear coordinate system becomes

(8) $$\frac{d^{2}{\hat{u}}_{z}}{d^{2}s}=\frac{\varepsilon (s)}{h}.$$

Assuming that the length of the neutral axis remains unchanged such that s≈x, the formulation simplifies to

(9) $$\frac{d^{2}{\hat{u}}_{z}}{d^{2}x}=\frac{\varepsilon (x)}{h}.$$

The simplification of an unchanged neutral axis length is applicable to a beam with clamped ends, and the curved displacement formulations are valid for such a case [11].

In this way, the curved displacement formulation extends the applicability of Ko’s displacement theory to geometrically nonlinear deformation states while retaining the strain-based reconstruction framework.

The derivation above assumes small displacement kinematics. This assumption is valid when

(10) $$|\frac{\delta u_{z}}{\delta x}|<<1.$$

For large structural deformations, nonlinear strain contributions become significant, meaning that additional nonlinear terms must be considered [32,34]. The nonlinear strain–displacement relation can then be expressed as

(11) $$\epsilon \left(x\right)=-h\frac{d^{2}u_{z}}{dx^{2}}+\frac{1}{2}{\left(\frac{du_{z}}{dx}\right)}^{2}.$$

The nonlinear contribution becomes increasingly important for post-buckled structures exhibiting large rotations and geometric nonlinearities.

### 2.3. Extension to Plate Structures

**Figure 4:**
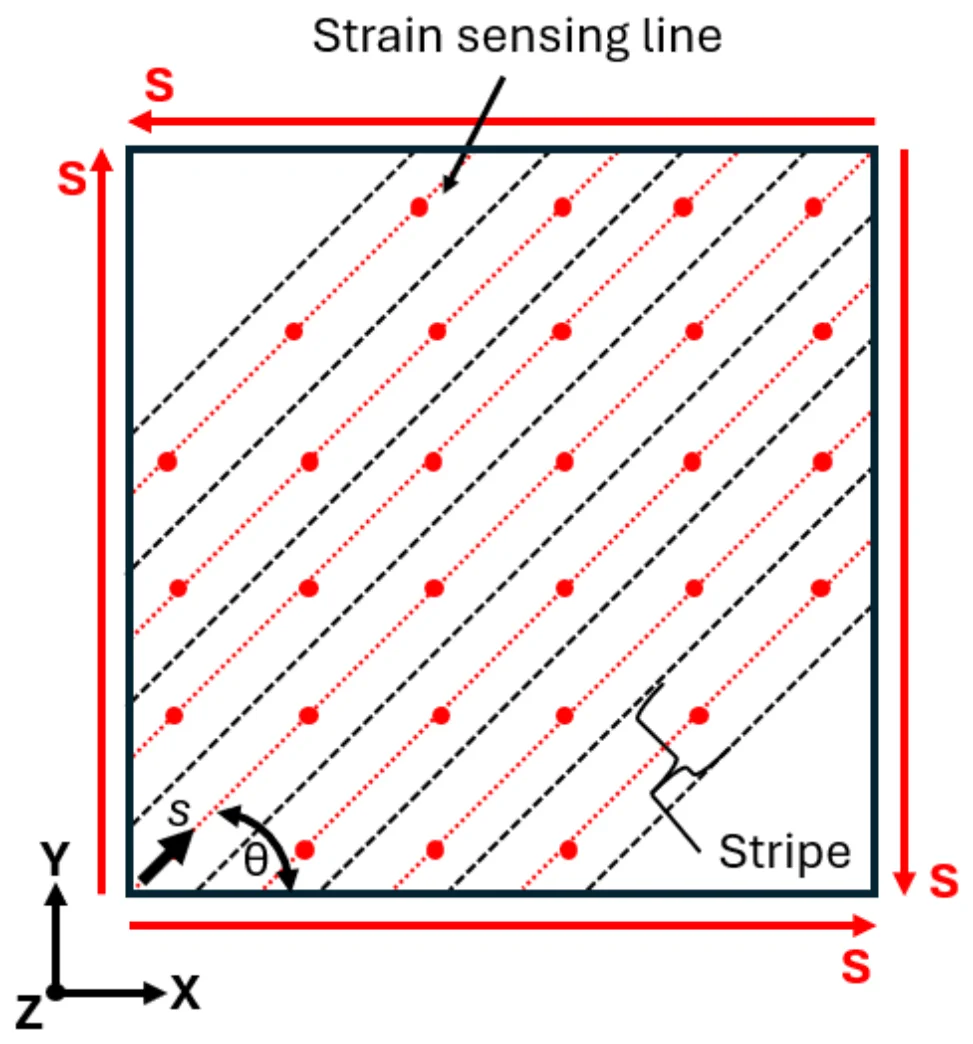
Division of a plate loaded by a shear force S into narrow strips for determination of the displacement.

The strain-based reconstruction methodology can be extended from beam structures to plates by decomposing the plate into multiple narrow strips, as illustrated in Figure 4. Each strip is treated as an equivalent beam aligned with a strain sensing line [11,28]. The strain sensing line is the line and direction along which the strain is measured with the DOFS. The directions of the strain sensing lines, and hence the strips, can be chosen and varied by the angle θ.

The strain field within the plate is described by the axial strain ϵ and shear strain γ:

(12) $$\begin{array}{cc} \varepsilon_{x}\left(x,y,z\right) & =\varepsilon_{x}^{0}\left(x,y\right)-h\cdot u_{z,xx}\left(x,y\right)\\ \varepsilon_{y}\left(x,y,z\right) & =\varepsilon_{y}^{0}\left(x,y\right)-h\cdot u_{z,yy}\left(x,y\right)\\ \gamma_{xy}\left(x,y,z\right) & =\gamma_{xy}^{0}\left(x,y\right)-h\cdot 2u_{z,xy}\left(x,y\right) \end{array}$$

where the superscript ^0^ denotes membrane strain contributions and the remaining terms correspond to bending strain contributions. Membrane strains cannot be neglected in plate structures because in-plane deformation contributes significantly to the overall strain field.

For a strain sensing line oriented at an angle θ, as shown in Figure 4, the measured strain $\varepsilon_{f}$ along the strain sensing line can be expressed as

(13) $$\varepsilon_{f}=\varepsilon_{f}^{0}+h\kappa_{f},$$

where the membrane strain contribution is

(14) $$\varepsilon_{f}^{0}=\varepsilon_{x}^{0}\cos^{2}\theta +\varepsilon_{y}^{0}\sin^{2}\theta +\gamma_{xy}^{0}\sin\theta \cos\theta$$

and the bending strain contribution becomes

(15) $$\kappa_{f}=u_{z,xx}\cos^{2}\theta +2u_{z,xy}\sin\theta \cos\theta +u_{z,yy}\sin^{2}\theta .$$

Introducing a local coordinate *s* along the sensing line,

(16) $$u_{z,f}\left(s\right)=u_{z}\left(x\left(s\right),y\left(s\right)\right),$$

leads to the curvature relation

(17) $$\frac{d^{2}u_{z,f}}{ds^{2}}=u_{z,xx}\cos^{2}\theta +2u_{z,xy}\sin\theta \cos\theta +u_{z,yy}\sin^{2}\theta =\kappa_{f}.$$

Substitution into Equation (13) then yields

(18) $$\varepsilon_{f}\left(s\right)=\varepsilon_{f}^{0}\left(s\right)+h\cdot u_{z,f}^{\prime \prime }\left(s\right)=\varepsilon_{f}^{0}\left(s\right)+h\kappa_{f}.$$

### 2.4. Application to Post-Buckled Shear Plates

**Figure 5:**
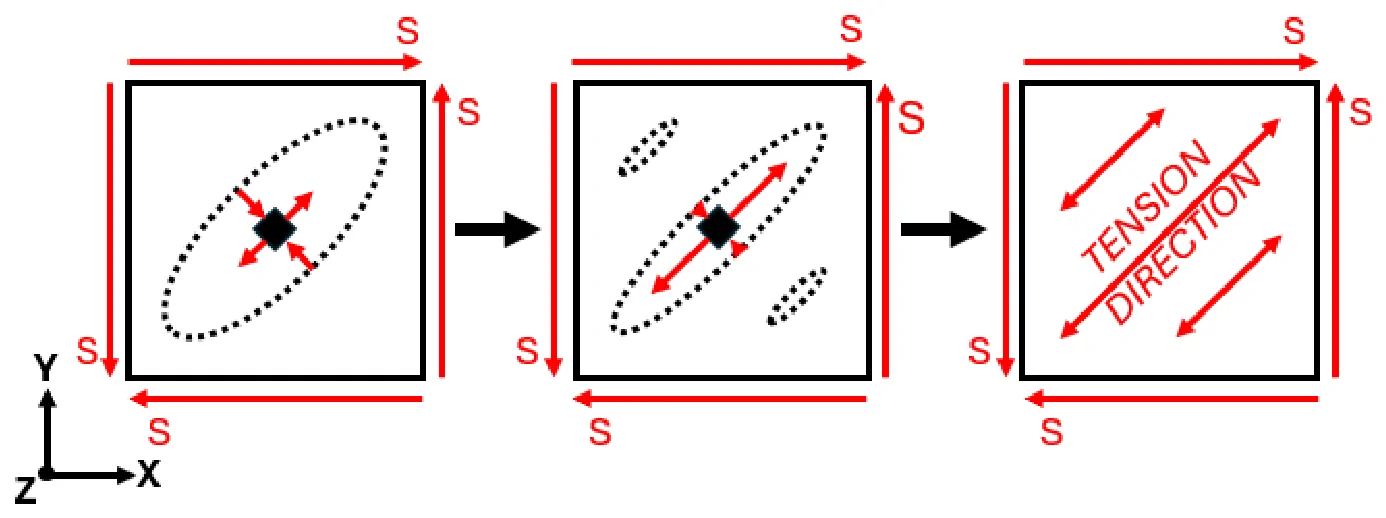
Development of tension diagonals in a post-buckled shear-loaded plate with the postbuckling wave shape indicated by the dashed lines.

The present work focuses on shear-loaded plates operating in the post-buckling regime. Such plates develop characteristic diagonal stress fields consisting of a tension diagonal, as illustrated in Figure 5.

The tension diagonal is dominated by membrane strains, whereas the diagonal normal to the tension diagonal exhibits significantly lower membrane strain contributions and stronger bending-dominated deformation behavior [35]. Consequently, strain sensing lines positioned along the compression diagonal primarily measure bending strain, which would simplify Equation (18) to the beam-based formulation previously introduced in Equation (5). This simplification forms the theoretical basis for applying Ko’s displacement theory to post-buckled shear-loaded plates and enables strain-based reconstruction of their out-of-plane deformation using DOFS.

## 3. Experimental Setup

The setup for the experimental tests using a Wagner beam is discussed in Section 3.1, while the outline of the testing is discussed in Section 3.2. Next, Section 3.3 presents the setup of the DOFS system to measure the strain data for the computation of the out-of-plane displacement. The DIC used for the displacement measurements is then explained in Section 3.4.

### 3.1. Wagner Beam

A plate girder consisting of thin shear-loaded plates connected through stiffeners and flanges is commonly referred to as a Wagner beam. The structural behavior of such beams has been extensively investigated in the context of lightweight aerospace structures [36,37,38,39,40]. Under increasing shear loading, the plates initially carry shear through approximately uniform tensile and compressive stress fields. Once the buckling shear load is exceeded, the plates enter the post-buckling regime and develop a characteristic diagonal deformation wave accompanied by a tension field. The post-buckling response of shear plates makes Wagner beams particularly suitable for investigating strain-based shape sensing approaches. The plates show the desired behavior that is needed in order to apply Ko’s displacement theory for a plate, as discussed in Section 2.4.

**Figure 6:**
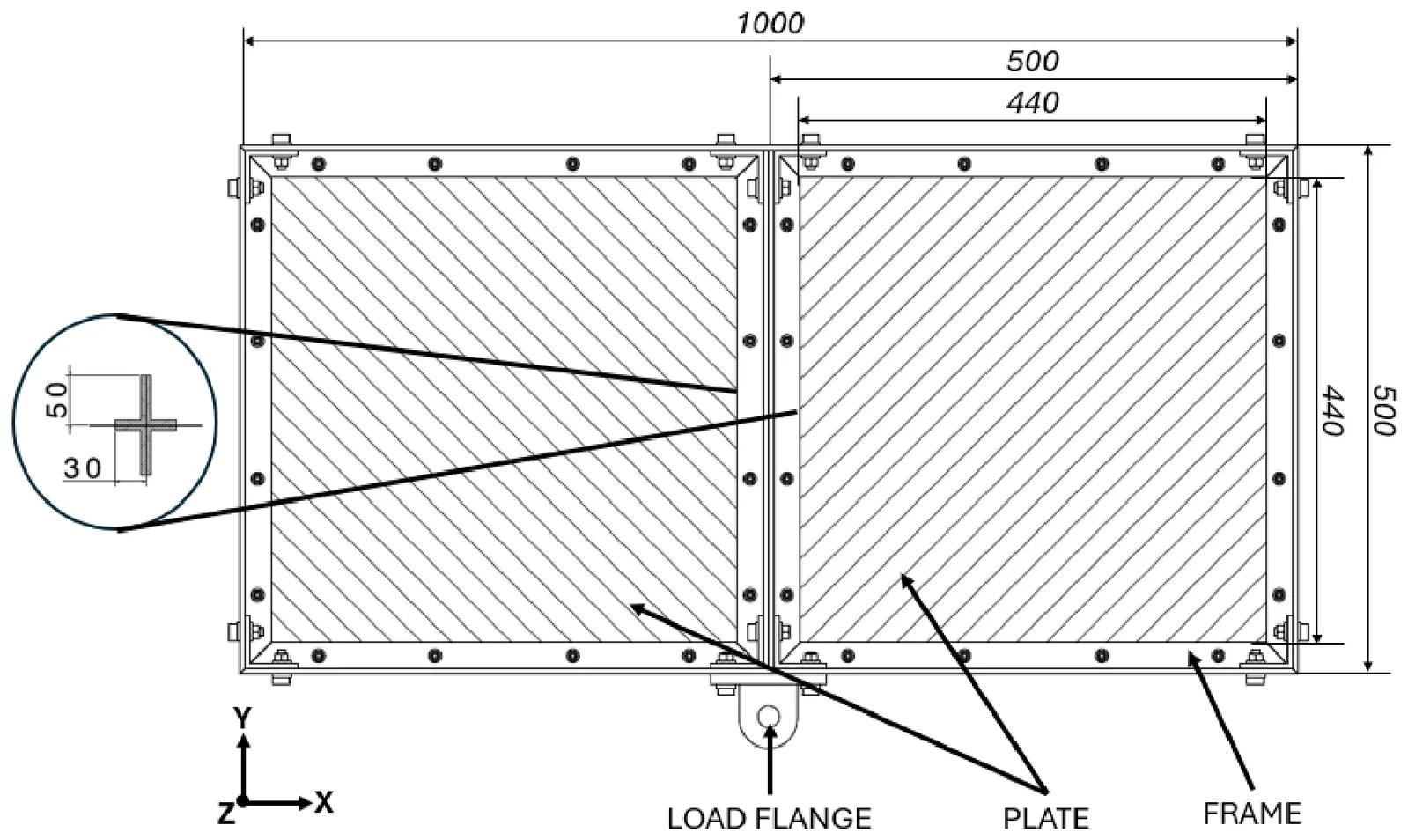
Wagner beam front view with cross-section of the frame–plate interface; dimensions are in mm.

In the present work, a dedicated Wagner beam test rig at the Department of Aerospace Science and Technology was used to investigate displacement reconstruction of post-buckled shear plates using DOFS and DIC. The experimental Wagner beam is shown in Figure 6.

The structure consists of two square shear-loaded plates connected through a stiffened aluminum frame system. Each plate has a side length of 500 mm, while the plate material and thickness can be adapted depending on the experimental campaign. The plates are surrounded by L-flanges made of aluminum alloy EN AW 6060 T6 with a thickness of $t_{Frame}=5$ mm. The smaller flange is connected to the plate, resulting in a free plate length of 440 mm. Neighboring frame segments are connected through bolts. In addition, a load introduction flange is mounted at the center of one longitudinal side of the Wagner beam to introduce the external loading. The selected arrangement produces opposite diagonal post-buckling deformation patterns in the two plates and promotes symmetric deformation behavior.

**Figure 7:**
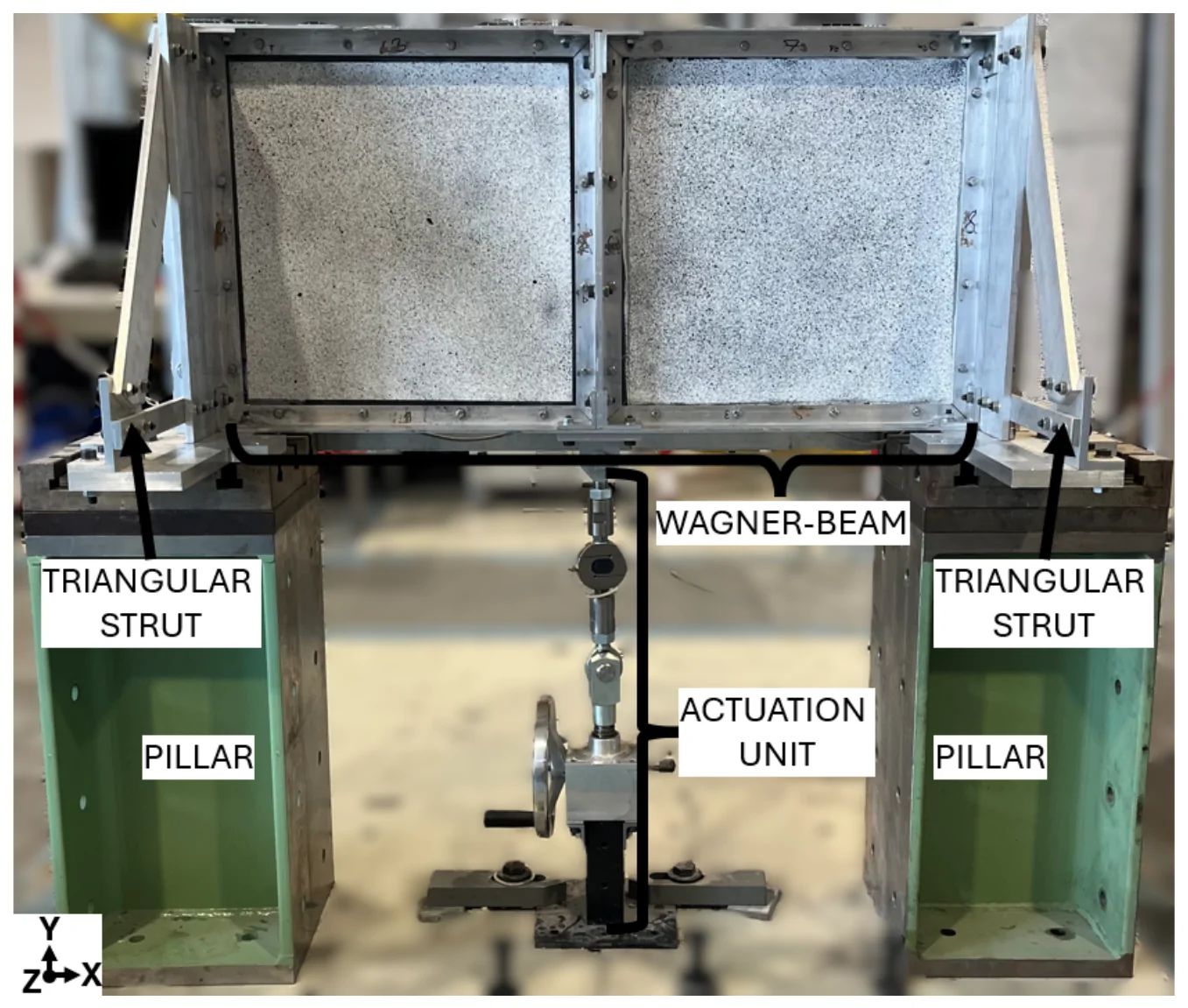
Experimental Wagner beam test rig setup.

The Wagner beam test rig is mounted on two rigid steel support pillars connected to the laboratory floor, as shown in Figure 7.

Positioning pins integrated into the support structure ensure repeatable alignment of the Wagner beam during installation. The beam is supported over a contact area of 100 mm ×50 mm on both sides, resulting in a free bending length of approximately 450 mm between the supports and the point of application of the central load.

A triangular strut is integrated into the fixture system to limit undesired out-of-plane rigid-body motion while allowing in-plane deformation of the Wagner beam. This strut system uses a triangular beam geometry applied to the front and back sides of the frame, providing adjustable lateral support against out-of-plane motion and reducing sensitivity to manufacturing and assembly imperfections.

The external load is applied via a manually operated screw jack connected to the central load-introduction flange. A load cell with a maximum capacity of 1000 kg is integrated into the loading path to measure the applied force. In addition, a linear variable differential transformer (LVDT) measures the vertical displacement at the point of load introduction. Both signals are synchronized and recorded using LabVIEW 18.0 software environment. The maximum admissible load without plastic deformation of the frame structure is 20 kN.

### 3.2. Loading Method and Strain Gauge Setup

The tests are conducted by quasi-static manual loading and unloading of the Wagner beam test rig, equipped with 1 mm thick aluminum alloy plates. A moderate loading rate is selected to avoid dynamic effects.

**Figure 8:**
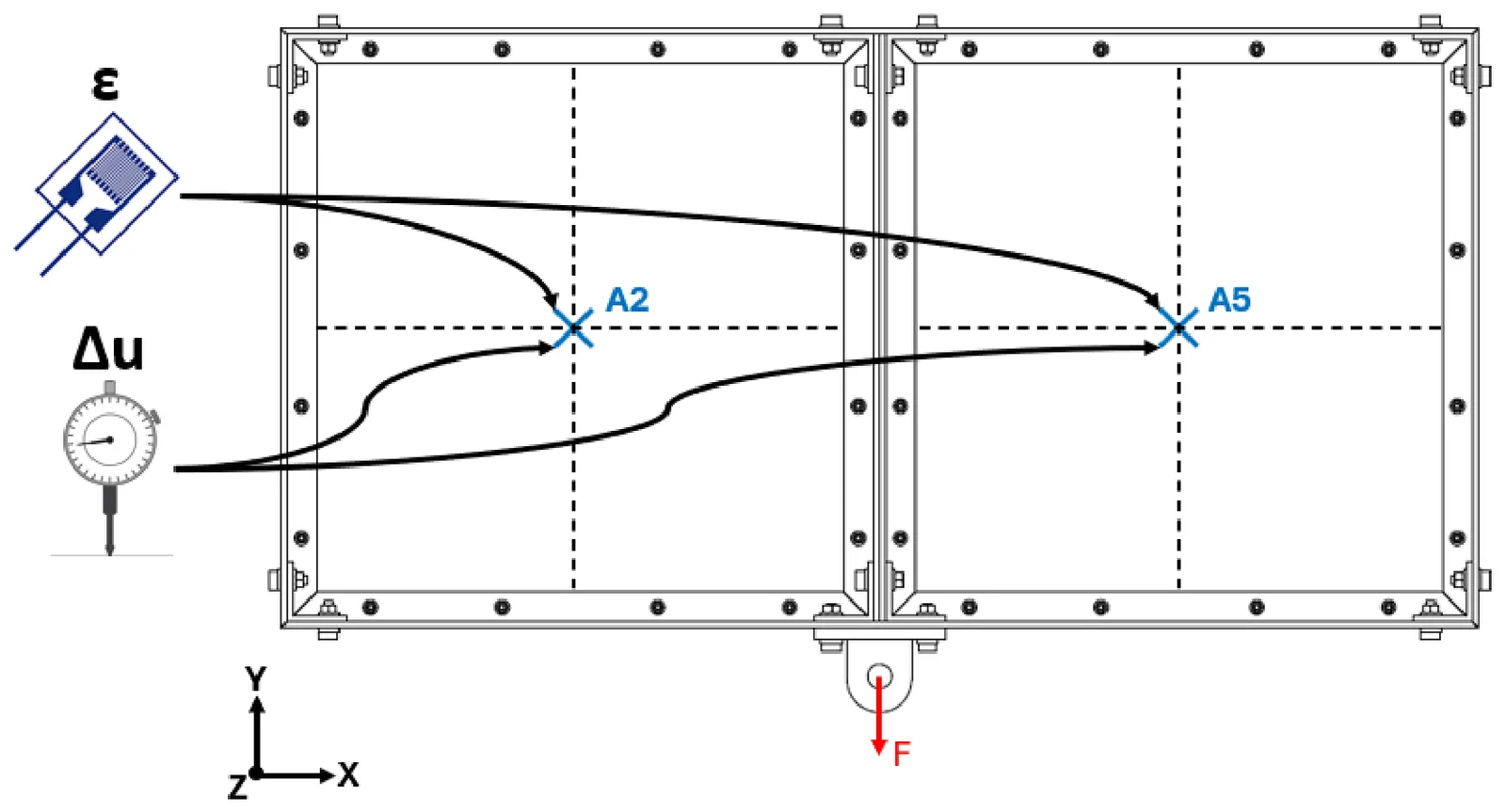
Application of strain gauge and dial indicator on the plates of the Wagner beam at positions A2 and A5.

To monitor the structural response during loading, back-to-back strain gauges (SGs) are installed at the center of each plate, as shown in Figure 8.

The SGs are oriented at an angle of 45° relative to the global x−y coordinate system, corresponding to the dominant diagonal deformation direction of the post-buckled shear plates. Using the front and back SG signals, the axial membrane strain component $\varepsilon_{axial}$ can be determined as follows:

(19) $$\varepsilon_{axial}=\frac{\varepsilon_{SG}+\varepsilon_{SG_{back}}}{2}=\frac{\varepsilon_{axial}+\varepsilon_{bending}+\varepsilon_{axial}-\varepsilon_{bending}}{2}$$

where the bending strain components cancel due to their opposite signs on the two plate surfaces.

In addition, dial indicators are positioned at the center of each plate to measure the out-of-plane displacement during loading. These measurements serve as an additional reference for validating displacement reconstruction approaches. The locations of the SGs and dial indicators are illustrated in Figure 8.

### 3.3. Distributed Optical Fiber Sensors

**Figure 9:**
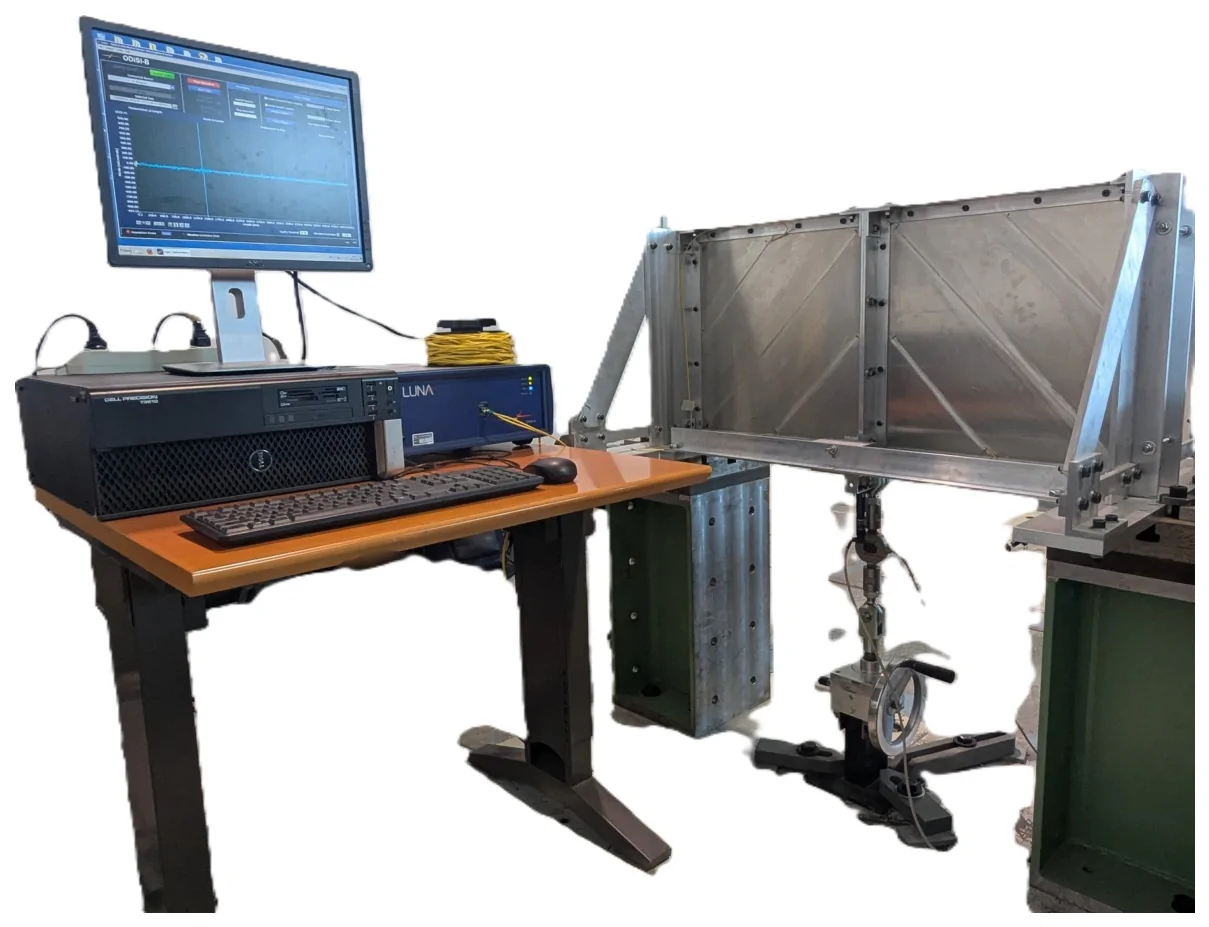
Optical fiber measurement system.

**Figure 10:**
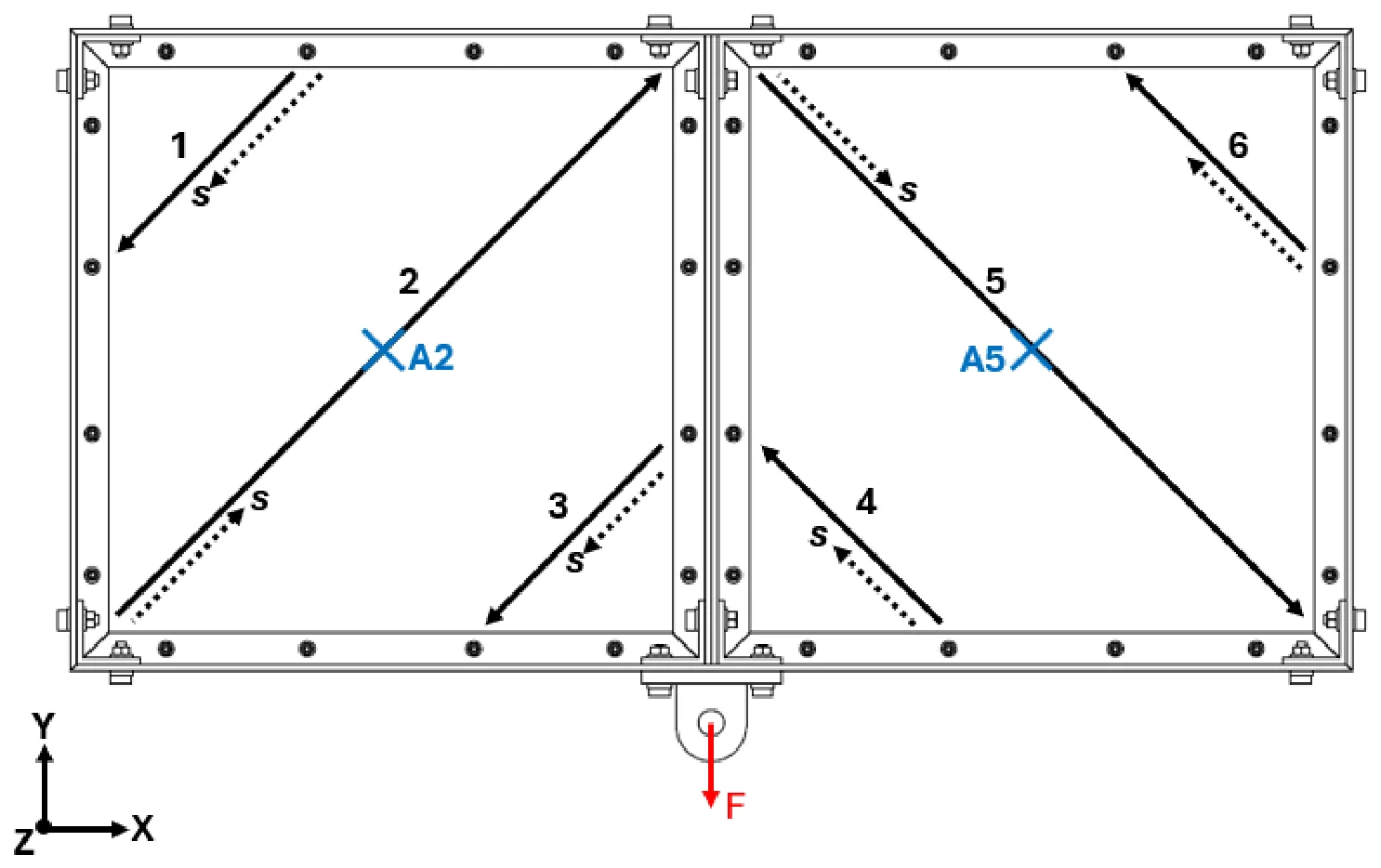
Definition of the fiber paths for the DOFS system. The *s* coordinate indicates the local fiber path direction.

Wagner beam plates are instrumented on one side with DOFS bonded directly onto the plate surface using an epoxy adhesive. These sensors consist of optical fibers with a numerical aperture of 0.41 and a polyimide coating according to user requirements. Strain is monitored along the beam diagonals and along two additional segments oriented parallel to the diagonals, which enhances sensitivity to buckling-induced bending. In total, six measurement paths are defined, three on each plate; the optical fiber is arranged accordingly to cover these six paths, as illustrated in Figure 9 and Figure 10.

The optical fiber enables strain measurement along its longitudinal axis. For this purpose, the ODiSI-B interrogator by Luna Innovations is employed. This device is a swept-wavelength interferometer capable of measuring the Rayleigh backscatter within optical fibers that originates from random fluctuations in the refractive index along the fiber length. Variations in strain or temperature relative to a reference state result in a spectral shift of the backscattered light, quantified as follows:

(20) $$\frac{\lambda -\lambda_{0}}{\lambda_{0}}=K_{T}\left(T-T_{0}\right)+K_{\varepsilon }\left(\varepsilon -\varepsilon_{0}\right)$$

where $K_{\varepsilon }=0.780$ and $K_{T}=6.45\;\times \;10^{-6}$ [1/°C] are respectively the strain and temperature sensitivities for the optical fiber used here [41,42], while $\lambda_{0}$, $T_{0}$, and $\varepsilon_{0}=0$ are respectively the wavelength, temperature, and strain at the initial reference state. The test involving the loading and unloading of the Wagner beam lasts only a minute, making it reasonable to assume that $T-T_{0}=0$, as follows:

(21) $$\varepsilon =\frac{\lambda -\lambda_{0}}{K_{\varepsilon }\lambda_{0}}.$$

The the computed strain is provided with a gauge length of 1.28 mm and a sampling frequency of 23.8 Hz. The optical fiber measurement system is synchronized with the load cell to capture data.

Following acquisition, the raw strain data are processed using MATLAB R2024a. The measured strain signals are corrected in order to isolate the bending strain component by subtracting the axial membrane strain contribution obtained from the back-to-back SG measurements located adjacent to fiber paths 2 and 5. In contrast, the total strain is used for the fiber paths 1, 3, 4, and 6. This is because no back-to-back SGs exist in the vicinity of those fiber paths, so no axial strain elimination can be conducted.

The bending strain along the fiber paths (see Figure 10) is then used as input for Ko’s displacement theory. Equation (7) applies the bending strains and calculates the out-of-plane displacement of the plate with respect to direction *s* along the fiber paths. The out-of-plane displacement calculation is repeated for each of the six fiber paths. The result is an out-of-plane displacement distribution along each fiber path. Because the plate is clamped into the frame of the Wagner beam, the boundary conditions for all six fiber paths were chosen as fully clamped regarding translational and rotational degrees of freedom.

### 3.4. Digital Image Correlation

The opposite side of the Wagner beam plates is instrumented using a DIC system for full-field displacement measurements. The system consists of two synchronized cameras arranged in a stereo configuration, and uses an LED light source. The DIC method evaluates displacement fields by tracking the movement of the surface pattern between subsequent digital images.

Full-field surface strain and out-of-plane displacement were measured using a stereo DIC system consisting of two ace2 cameras from BASLER, with an IMX545 sensor and a resolution of 12.3 MP. These cameras are equipped with JADE C-Mount lenses from Schneider Kreuznach with a 25 mm focal length and an aperture of 2.8. The cameras were positioned at a stereo angle of 25° with a distance of 2 m to the Wagner beam. The optical setup, including lens selection and camera distance, was adjusted to maximize spatial resolution over the selected measurement area. A monochromatic blue light Blue-X-Focus v3 was selected to improve measurement accuracy and suppress glare.

The system was calibrated using calibration targets with 13.35 mm spacing and a calibration residual of 0.044. Images were acquired at 10 Hz with VIC SNAP 10 software. Images were processed in VIC 3D 10 using a subset size of 29 px and a zero-normalized squared-difference correlation criterion with optimized 8-tap interpolation. The images show the out-of-plane displacement field $u_{z}$ across the plates of the Wagner beam during the entire duration of the force application.

The global field of view of the DIC system covers the entire Wagner beam structure, corresponding to approximately 1000 mm × 500 mm, including the surrounding frame. The average speckle diameter is selected from 0.75 mm to 1.25 mm for the random speckle pattern, corresponding to approximately 3–5 pixels per speckle.

To ensure the reliability of the stereo DIC system, the potential impact of the out-of-plane displacement on measurement uncertainty must be addressed. In the present study, this impact is minimized by two primary factors: the stereo geometry, and the scale of out-of-plane displacement relative to the focal setup. With a stable stereo angle of 25° and a sub-pixel calibration residual of 0.044 pixels, the system maintains robust spatial tracking across all three dimensions; moreover, the maximum out-of-plane displacement of the plate is less than 4 mm, which is minor compared to the camera-to-specimen working distance of 2000 mm. The resulting out-of-plane scale error is estimated to be well within the baseline system noise, confirming that the displacement comparisons remain accurate.

**Figure 11:**
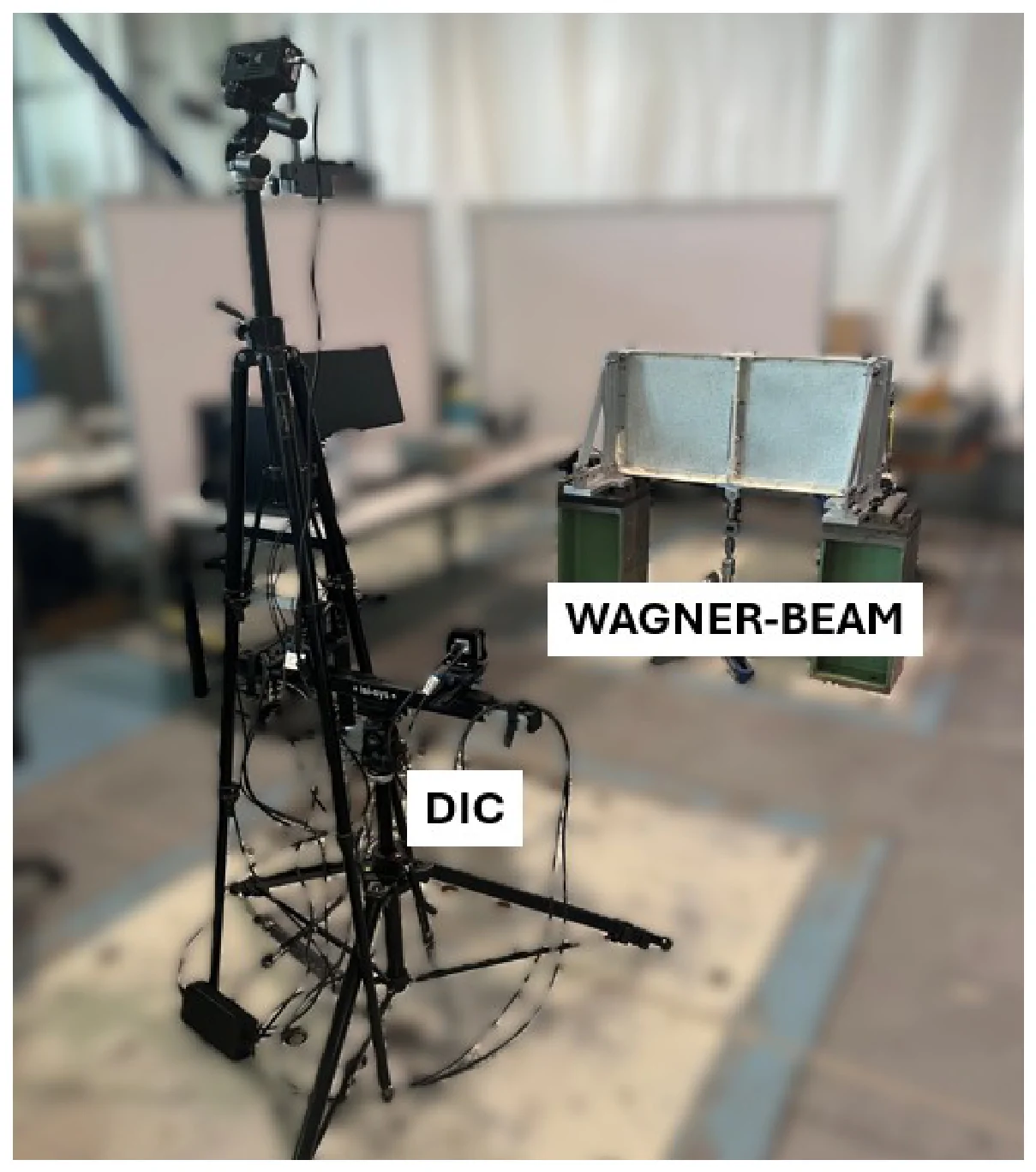
DIC measurement system used for full-field displacement acquisition.

The positioning of the DIC system relative to the Wagner beam test rig is shown in Figure 11. The DIC, DOFS, load cell, and displacement measurements are synchronized to enable direct comparison between strain-based displacement reconstruction and optically measured full-field displacements.

## 4. Results

This section presents the validation of the Wagner beam test rig and the applied measurement techniques in Section 4.1. Following the validation phase, Section 4.2 presents the analysis and discussion of the out-of-plane displacement fields captured during the experimental campaign using both DIC and DOFS measurements.

### 4.1. Test Rig Setup Assessment

**Figure 12:**
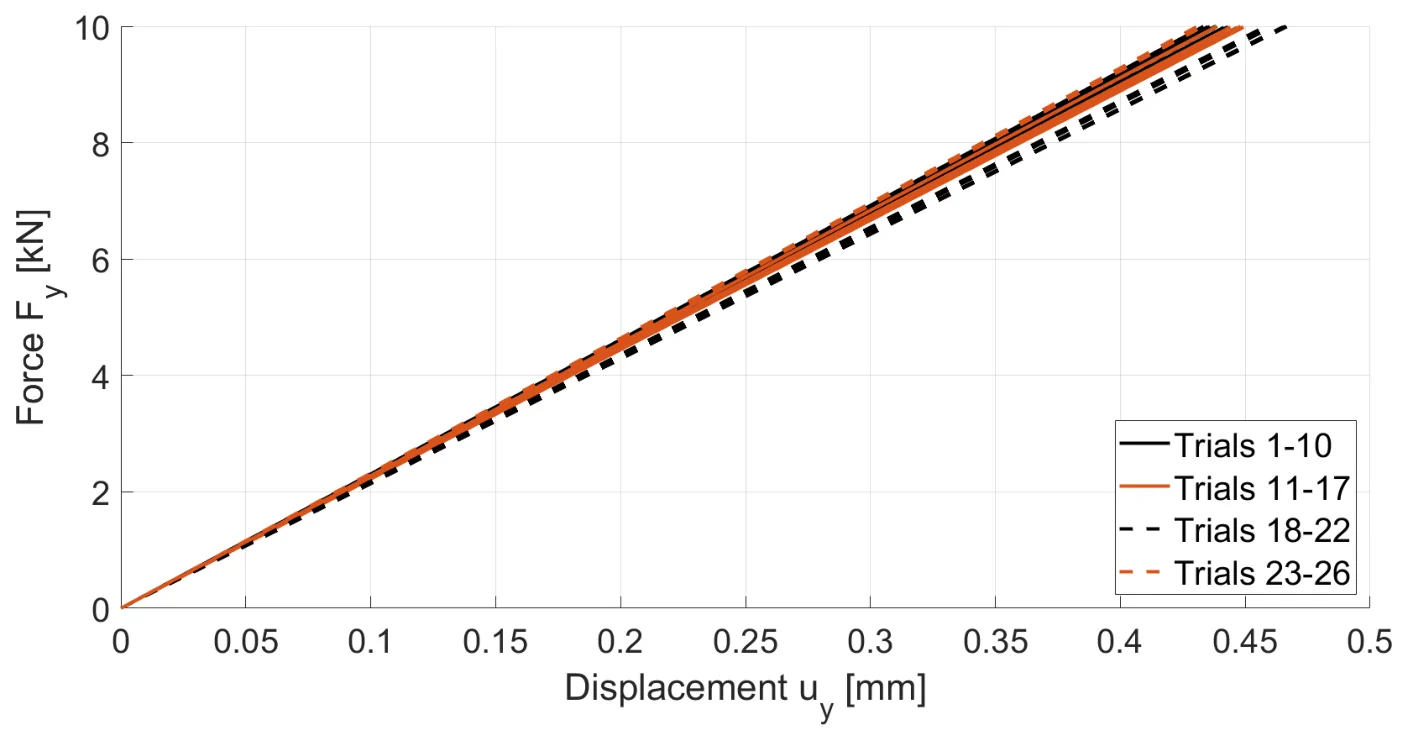
Force–displacement ($F_{y}-u_{y}$) graph for 26 trials of the experimental campaign.

The repeatability and compliance of the Wagner beam test rig are evaluated using the relationship between the applied load $F_{y}$ and the resulting downward displacement $u_{y}$ at the load introduction point. The experimental campaign was conducted in four trial batches. The Wagner beam remained mounted in the test rig within each batch, and was removed from the mounting structure between batches. The curves for the force $F_{y}$ to displacement $u_{y}$ at the load introduction point of the Wagner beam are shown in Figure 12 for all 26 trials across the four trial batches.

At an applied load of 10 kN, the measured downward displacement $u_{y}$ at the load introduction point ranges between 0.43 mm and 0.46 mm, corresponding to a maximum variation of approximately 6.5%. These results indicate good repeatability of the experimental setup despite the remounting operations between trial batches.

The validation of the Wagner beam test rig setup is assessed by comparing the DIC measurements of strain ε and out-of-plane displacement $u_{z}$ at the center point of the plates (see Figure 8) with measurements from a dial indicator and SGs. For trials 1–4, the displacements and strains are reported in Table 1.

Comparison of the out-of-plane displacement $u_{z}$ shows that DIC and dial indicator measurements are in close agreement, with a maximum deviation of 4.7%. Comparison of the strain ε between the SG and DIC shows an error lower than 21.7%. With the exception of two values, the error is below 8.4%. The error is computed by comparing the dial indicator or SG measurement to the DIC measurement, as the following example shows for the strain measurement:

(22) $$Error=(1-\frac{\varepsilon_{SG}}{\varepsilon_{DIC}})*100.$$

The applicability of the assumption of small displacement in Ko’s displacement theory is assessed using the displacement gradient criterion. For the investigated configuration, the maximum gradient is as follows:

(23) $$|\frac{\delta u_{z}}{\delta x}|=0.028rad<<0.1rad$$

which confirms that the displacement gradients remain within the small slope range for the evaluated deformation state.

The DOFS strain measurements are validated by comparison with SG measurements at the center of the plates, as shown in Table 2.

**Figure 13:**
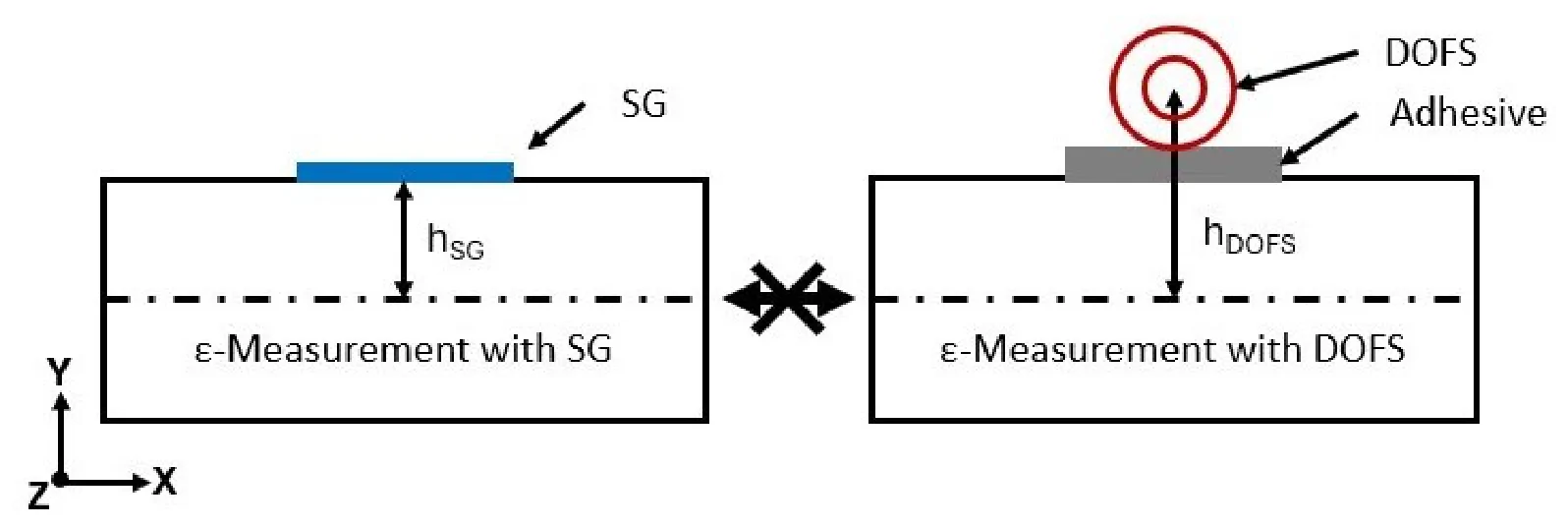
Influence of adhesive layer thickness on the effective distance between optical fiber and plate surface.

The initial comparison reveals a discrepancy for fiber path 2, whereas fiber path 5 shows good agreement. This difference is attributed to the different distances of the DOFS and SG sensing locations from the plate mid-plane. Due to the finite thickness of the adhesive layer and the optical fiber, the DOFS sensing axis is located farther from the plate mid-plane than the SG sensing location, as illustrated in Figure 13. Consequently, for the same local curvature, the DOFS measures a larger bending strain than the SG.

Because the bending strain varies linearly through the thickness, the DOFS strain can be corrected to the same distance from the neutral axis as the SG measurement. First, the curvature at the location of the SG is computed from the bending strain measured by the SG:

(24) $$\kappa_{SG}=\frac{\varepsilon_{bending,SG}}{h_{SG}}$$

The curvature at the location of the SG has to be identical to the curvature at the location of the DOFS:

(25) $$\kappa_{SG}=\kappa_{DOFS}=\kappa .$$

The effective distance $h_{DOFS}$ at the location of the DOFS is calculated with respect to the mid-plane of the plate:

(26) $$h_{DOFS}=\frac{\varepsilon_{DOFS}-\varepsilon_{axial}}{\kappa }.$$

The effective distance determined at the strain gauge reference location is assumed to be constant along the corresponding fiber path; therefore, local variations in adhesive thickness or fiber position are not individually corrected, and may affect the reconstructed displacement amplitude and profile through the strain-to-curvature conversion. This effective distance $h_{DOFS}$ is used to calculate the strain $\varepsilon_{DOFS,h_{SG}}$ which acts on the DOFS at a distance $h_{SG}$ from the mid-plane (Equation (27)). This enables comparison of the strain measurements by DOFS and SG without the influence of the adhesive layer:

(27) $$\varepsilon_{DOFS,h_{SG}}=\varepsilon_{DOFS}-\kappa \left(h_{DOFS}-h_{SG}\right).$$

After correction, the comparison between SG and DOFS measurements shows deviations of less than 4.1% for fiber paths 2 and 5, confirming good agreement between the two strain measurement systems. The corresponding error is computed as

(28) $$Error=(1-\frac{\varepsilon_{SG}}{\varepsilon_{DOFS_{h_{SG}}}})*100.$$

### 4.2. Out-of-Plane Displacement

The out-of-plane displacement is obtained by the methods of Section 3.3 and Section 3.4 in two different ways. The DIC calculates full-field out-of-plane displacement $u_{z}$, while the DOFS calculates the out-of-plane displacement distribution $u_{z}$ along the specified fiber paths.

**Figure 14:**
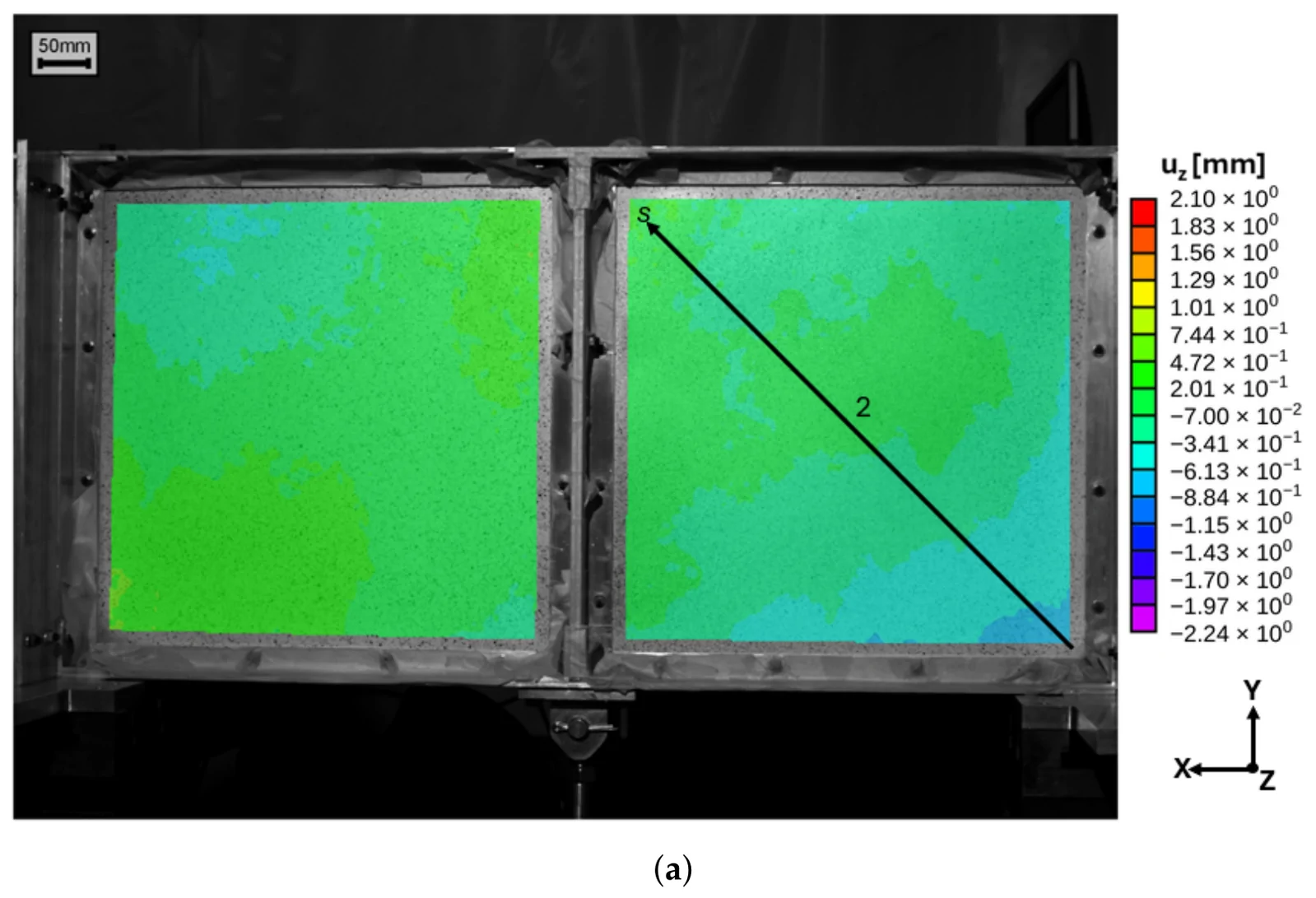

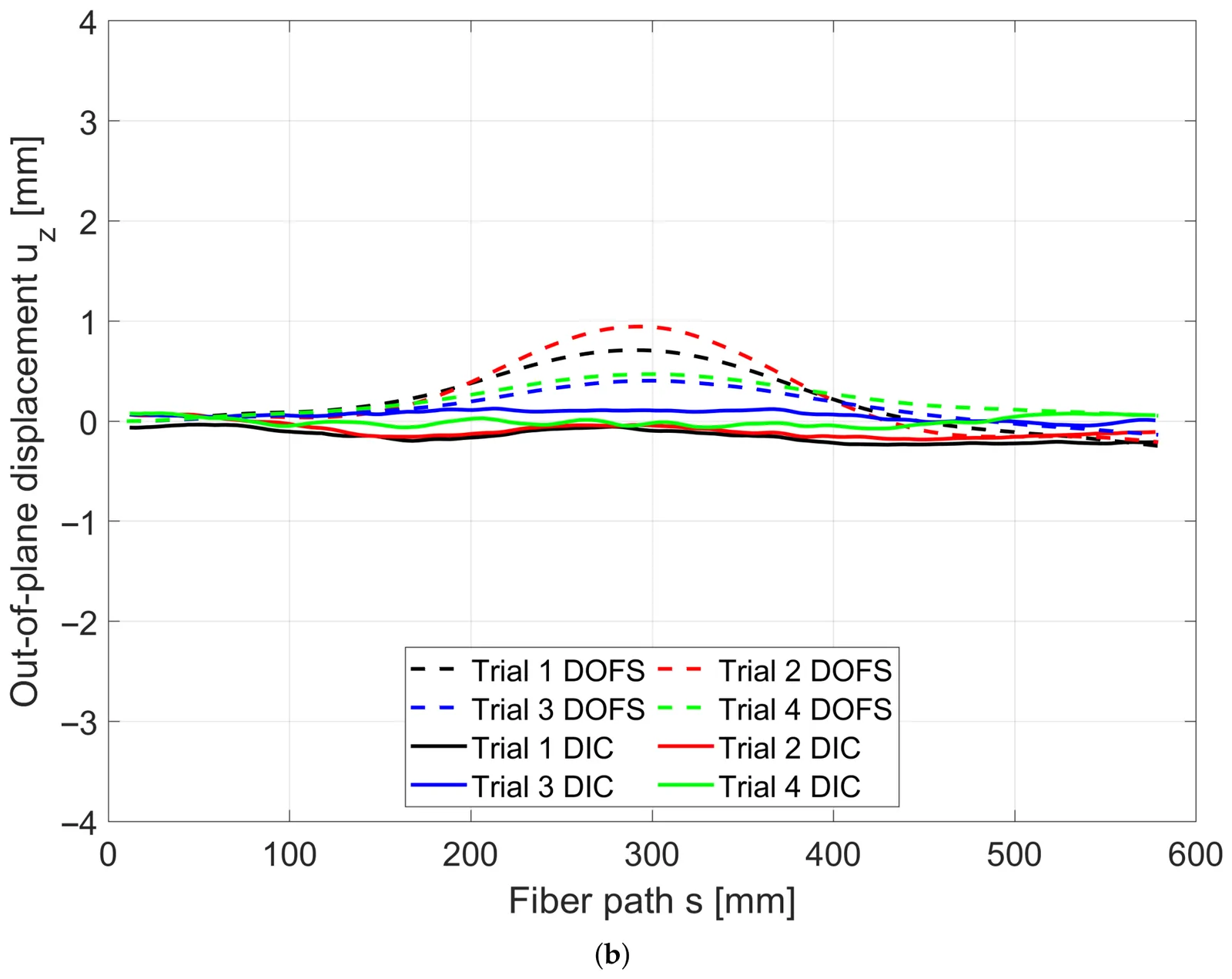
Out-of-plane displacement $u_{z}$ measured by DIC for both plates (**a**) and out-of-plane displacement $u_{z}$ along fiber path 2 by DIC and DOFS (**b**) at 2 kN loading.

**Figure 15:**
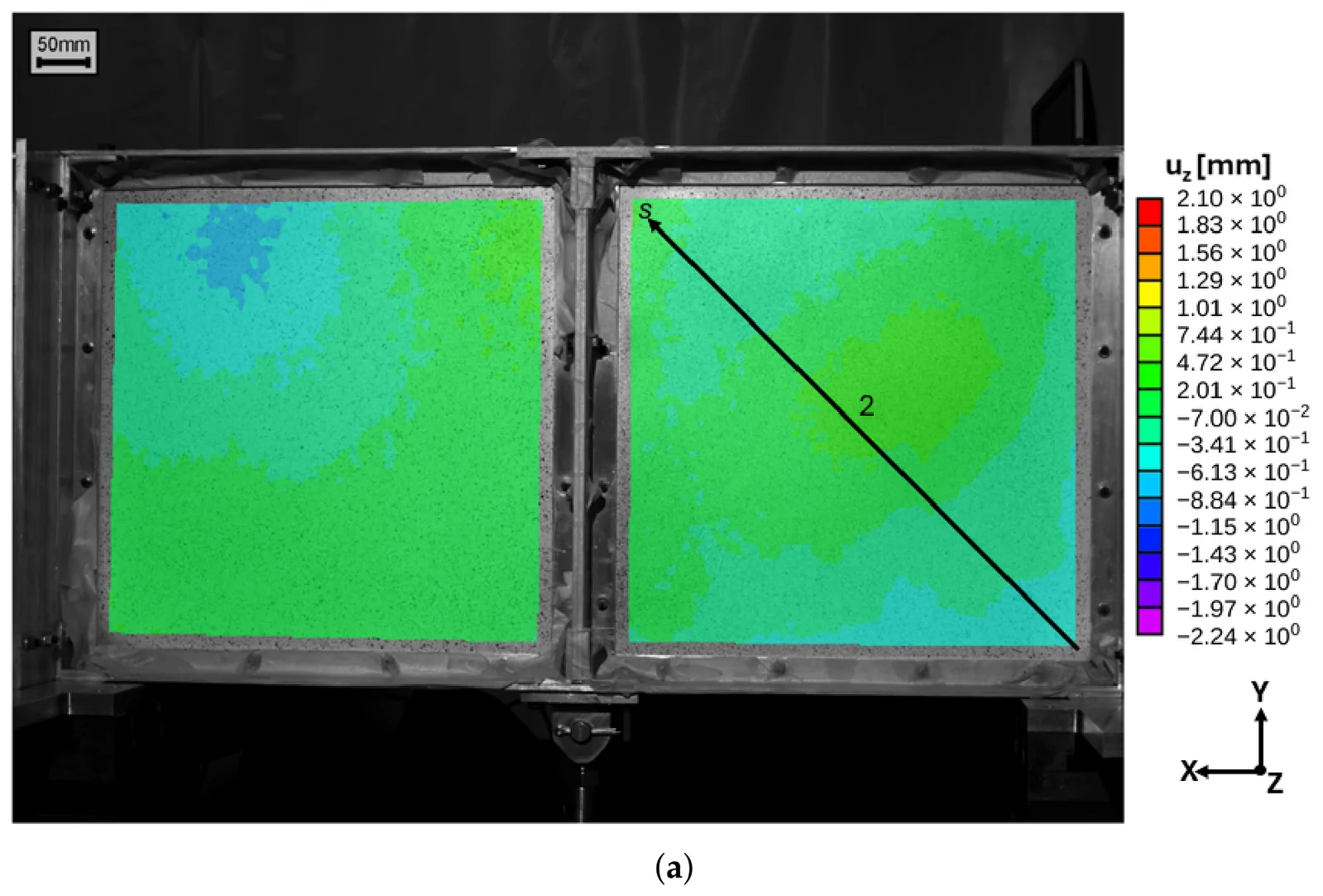

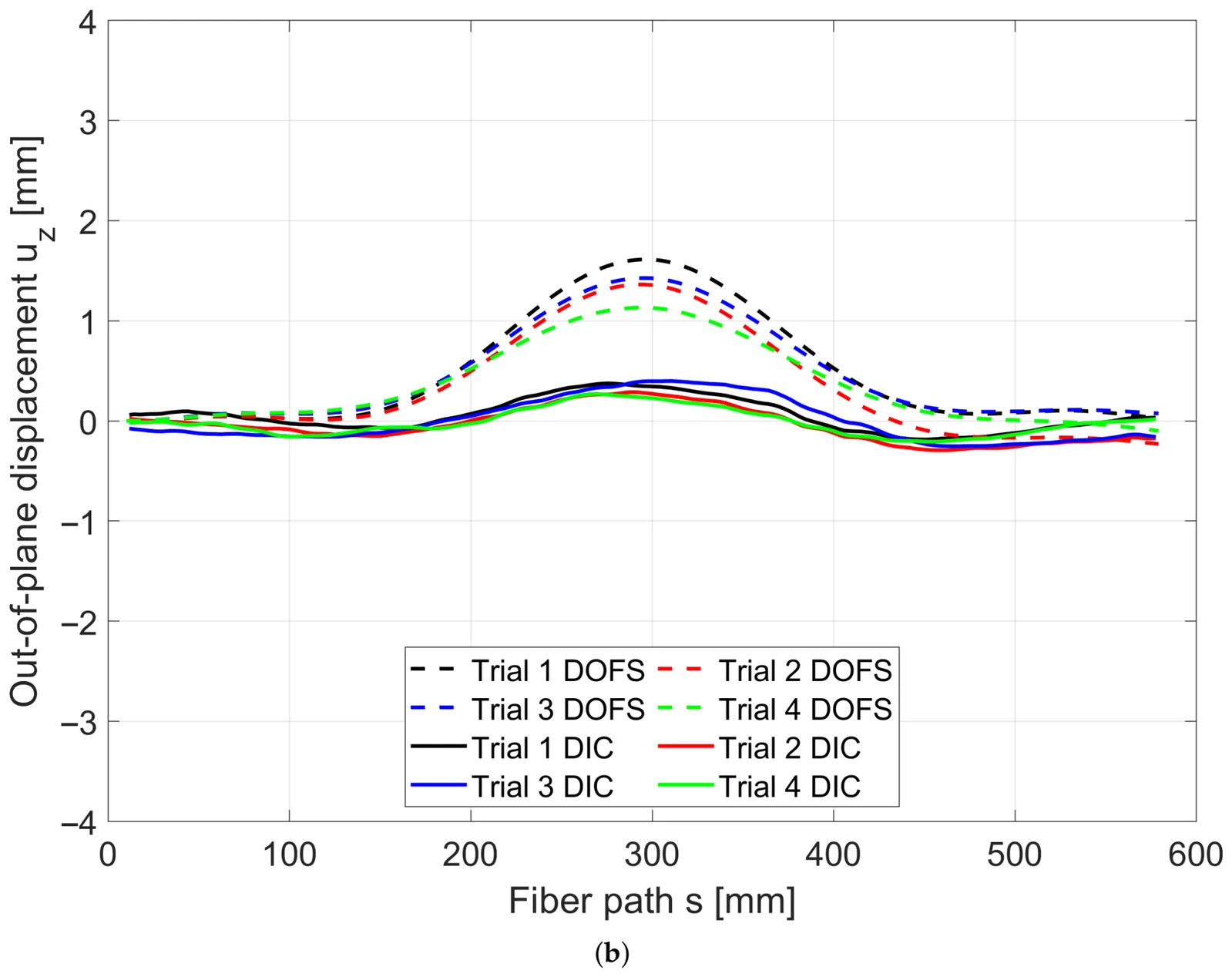
Out-of-plane displacement $u_{z}$ measured by DIC for both plates (**a**) and out-of-plane displacement $u_{z}$ along fiber path 2 by DIC and DOFS (**b**) at 4 kN loading.

**Figure 16:**
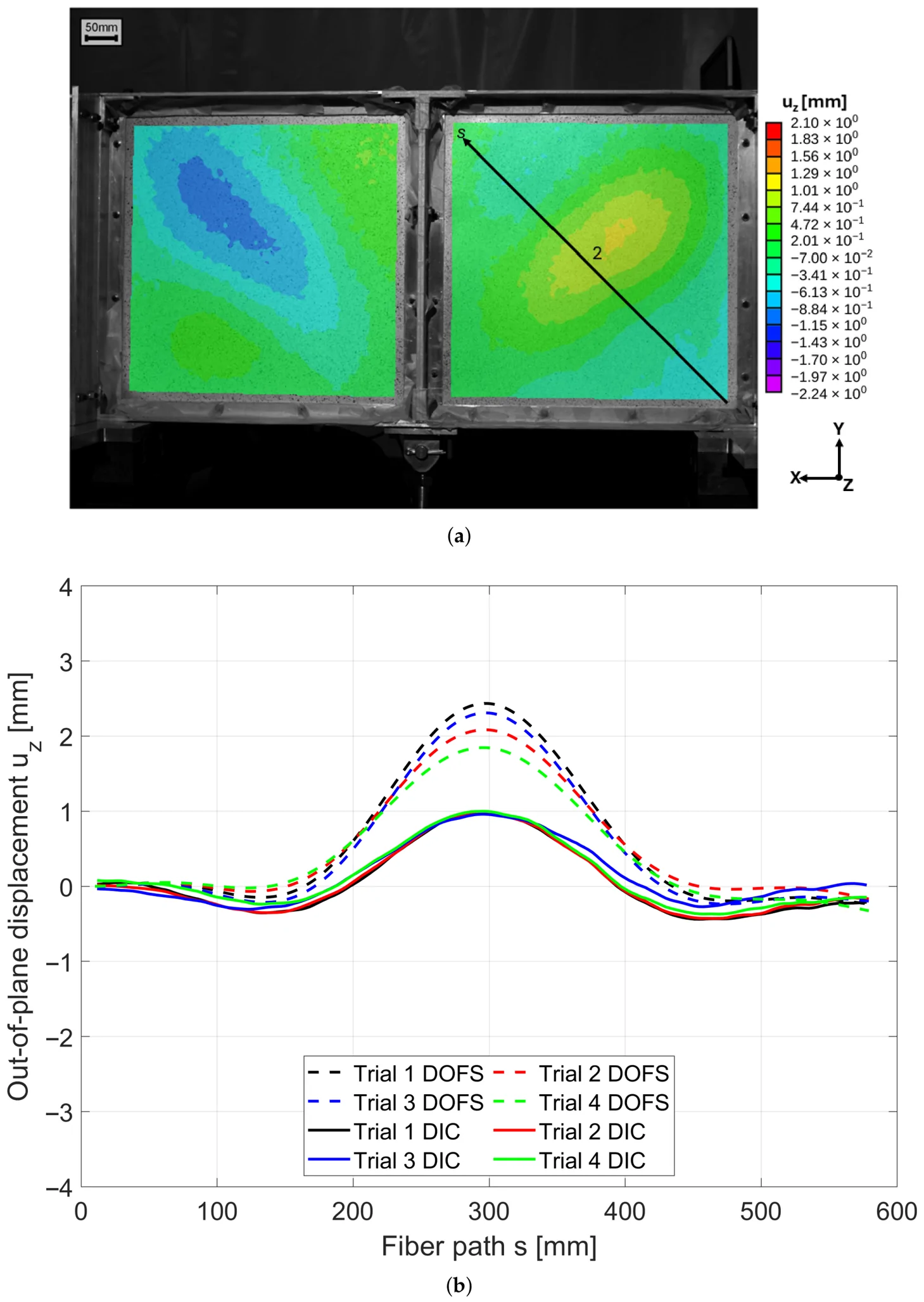
Out-of-plane displacement $u_{z}$ measured by DIC for both plates (**a**) and out-of-plane displacement $u_{z}$ along fiber path 2 by DIC and DOFS (**b**) at 6 kN loading.

**Figure 17:**
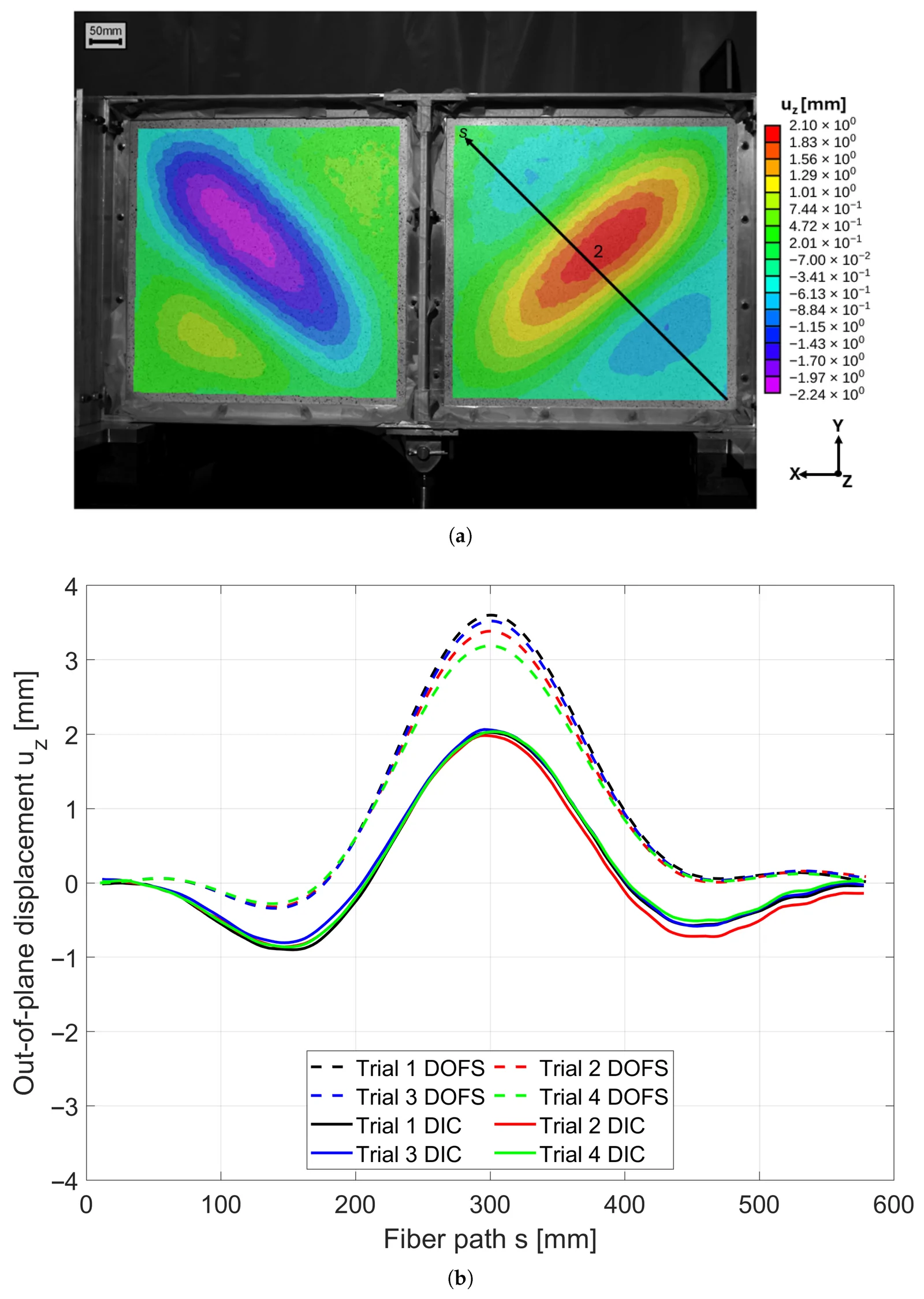
Out-of-plane displacement $u_{z}$ measured by DIC for both plates (**a**) and out-of-plane displacement $u_{z}$ along fiber path 2 by DIC and DOFS (**b**) at 10 kN loading.

Figure 14, Figure 15, Figure 16 and Figure 17 present the out-of-plane displacement $u_{z}$ fields of the DIC and the out-of-plane displacement $u_{z}$ distribution along fiber path 2 on the left plate of the Wagner beam for increasing applied forces. Figure 14a, Figure 15a, Figure 16a and Figure 17a present the out-of-plane displacement $u_{z}$ by the DIC, with the location and direction of fiber path 2 sketched into it. Figure 14b, Figure 15b, Figure 16b and Figure 17b present the comparison of the out-of-plane displacement $u_{z}$ distribution along fiber path 2 by DIC and DOFS.

At an applied force of 2 kN, the DIC measurements indicate only minor out-of-plane displacement $u_{z}$ with amplitudes below $u_{z}<0.2$ mm, as shown in Figure 14. In contrast, the out-of-plane displacement $u_{z}$ calculated by the DOFS shows a localized deformation with a maximum displacement between $u_{z}=0.5$ mm and $u_{z}=1$ mm near the center along fiber path 2.

Similar behavior is observed at an applied force of 4 kN, as shown in Figure 15.

While the DIC measurements still indicate relatively small and smooth deformation fields for the whole plates of the Wagner beam, the DOFS shows further increasing amplitudes for the out-of-plane displacement $u_{z}$ along the full fiber path 2 compared to the applied force of 2 kN.

The discrepancy between the pre-buckling and early post-buckling regimes is attributed to the assumptions underlying Ko’s displacement theory. The reconstruction approach requires bending strain as input. For paths 2 and 5, the membrane contribution estimated from the back-to-back SG measurements is subtracted from the measured DOFS strain. For paths 1, 3, 4, and 6, the total strain is used under the assumption that the membrane contribution is small relative to bending strain along the sensing direction. This assumption is distinct from the neglect of transverse shear deformation inherent in the Euler–Bernoulli formulation. Before the formation of the typical post-buckling shear wave for a shear-loaded plate, the strain field of the shear-loaded plate still contains significant shear contributions; consequently, the assumptions required for the out-of-plane displacement calculation with DOFS based on Ko’s displacement theory are only partially satisfied within this regime.

Figure 16 shows the out-of-plane displacement $u_{z}$ at an applied force of 6 kN.

Both plates develop the characteristic post-buckling shear wave consisting of one primary diagonal wave and two secondary smaller diagonal waves. In this regime, the out-of-plane displacement $u_{z}$ measurement based on DOFS captures the same global deformation characteristics as the DIC measurements, including the location of the primary and secondary wave peaks. However, the $u_{z}$ displacement amplitudes calculated by DOFS strain data remain 80–140% greater than those measured by DIC for the primary diagonal wave. The $u_{z}$ displacement amplitudes calculated by DOFS strain data for the secondary diagonal wave remain 0–100% greater than those measured by DIC.

The maximum investigated applied force of 10 kN is shown in Figure 17 as the resulting out-of-plane displacement $u_{z}$.

Both measurement methods, DIC and DOFS, consistently predict the same set of primary and secondary diagonal waves in the post-buckled plate. The locations of the peaks of the primary and secondary waves along fiber path 2 also coincide. Nevertheless, the out-of-plane displacement $u_{z}$ based on the DOFS strain data overestimates the amplitudes of the primary diagonal wave by approximately 60–75%. In contrast, the amplitudes of out-of-plane displacement $u_{z}$ of the secondary diagonal wave are underpredicted by 60% using DOFS as compared to DIC.

**Figure 18:**
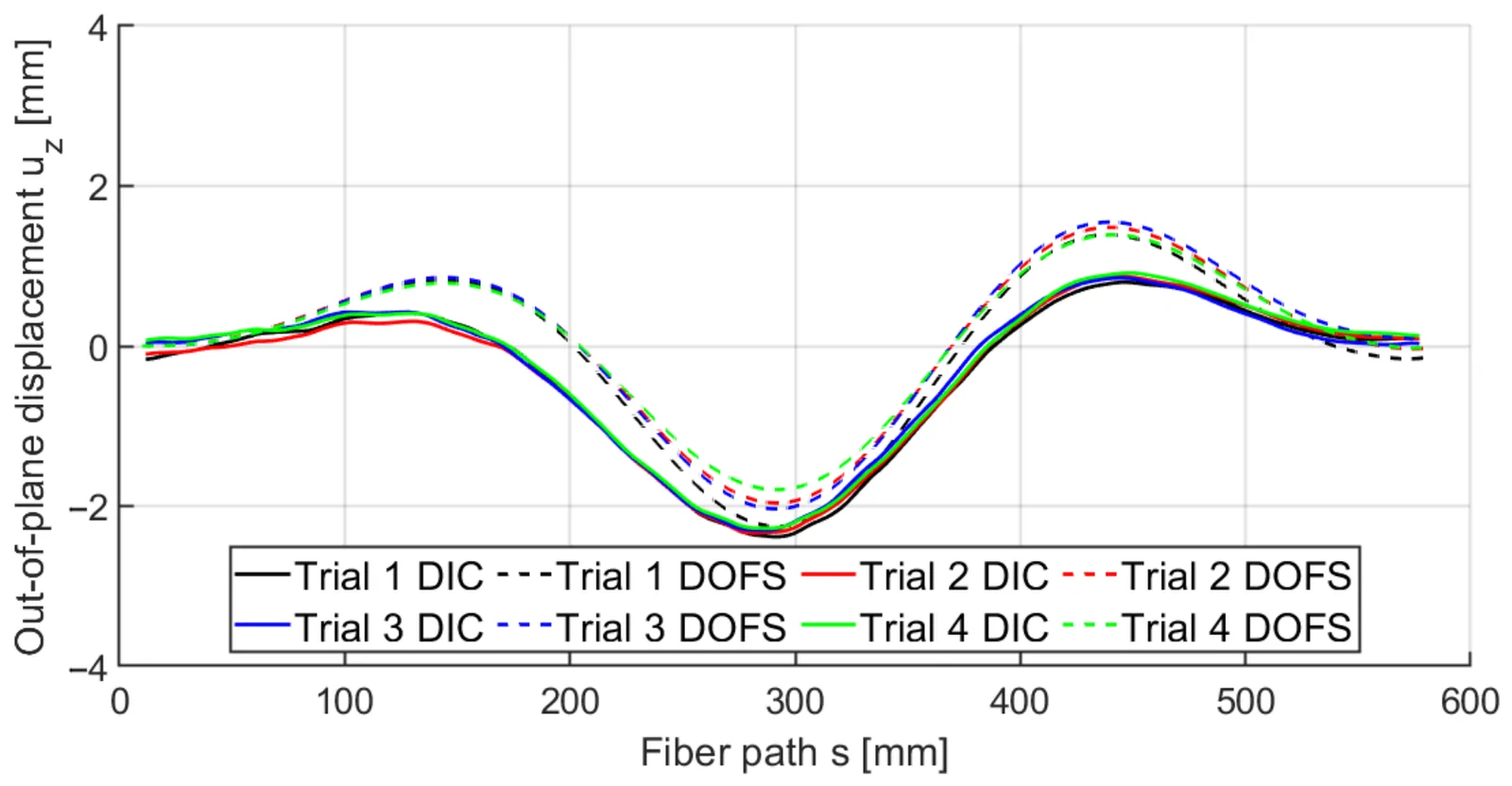
Out-of-plane displacement $u_{z}$ measured by DIC and calculated from DOFS data at 10 kN applied force for fiber path 5.

Figure 18 shows the out-of-plane displacement $u_{z}$ along fiber path 5 at an applied force of 10 kN for Trials 1–4.

The out-of-plane displacement $u_{z}$ for fiber path 5 calculated by both methods agrees regarding the amplitude and location of the primary diagonal wave of the post-buckled plate, with a maximum deviation of 25% between both methods; however, differences persist in predicting the secondary-diagonal wave amplitudes, with the out-of-plane displacement $u_{z}$ overpredicted by 60–80% using the DOFS method compared to the DIC method.

The differences between fiber paths 2 and 5 indicate the sensitivity of the shape reconstruction approach to local manufacturing and assembly imperfections, including fiber alignment, adhesive layer uniformity, and bonding quality [43]. Since the optical fibers are manually positioned and bonded to the plate surface, local variations in the sensing direction and distance from the neutral axis affect the accuracy of shape reconstruction. In future investigations, each of these aspects should be examined separately in order to determine the magnitude of influence for each effect. Especially for alignment and directional effects, a machine-based application of optical fibers could improve the quality of the results. Embedding the fibers within the plates, especially in composite plates, would minimize the effects of imperfections in bonding quality and adhesive layer uniformity. Although the corrected DOFS strains show good agreement with the SG measurements at the reference locations, residual effects of non-uniform adhesive thickness and fiber misalignment along the sensing paths cannot be distinguished quantitatively from errors associated with applying Ko’s displacement theory to the present configuration.

**Figure 19:**
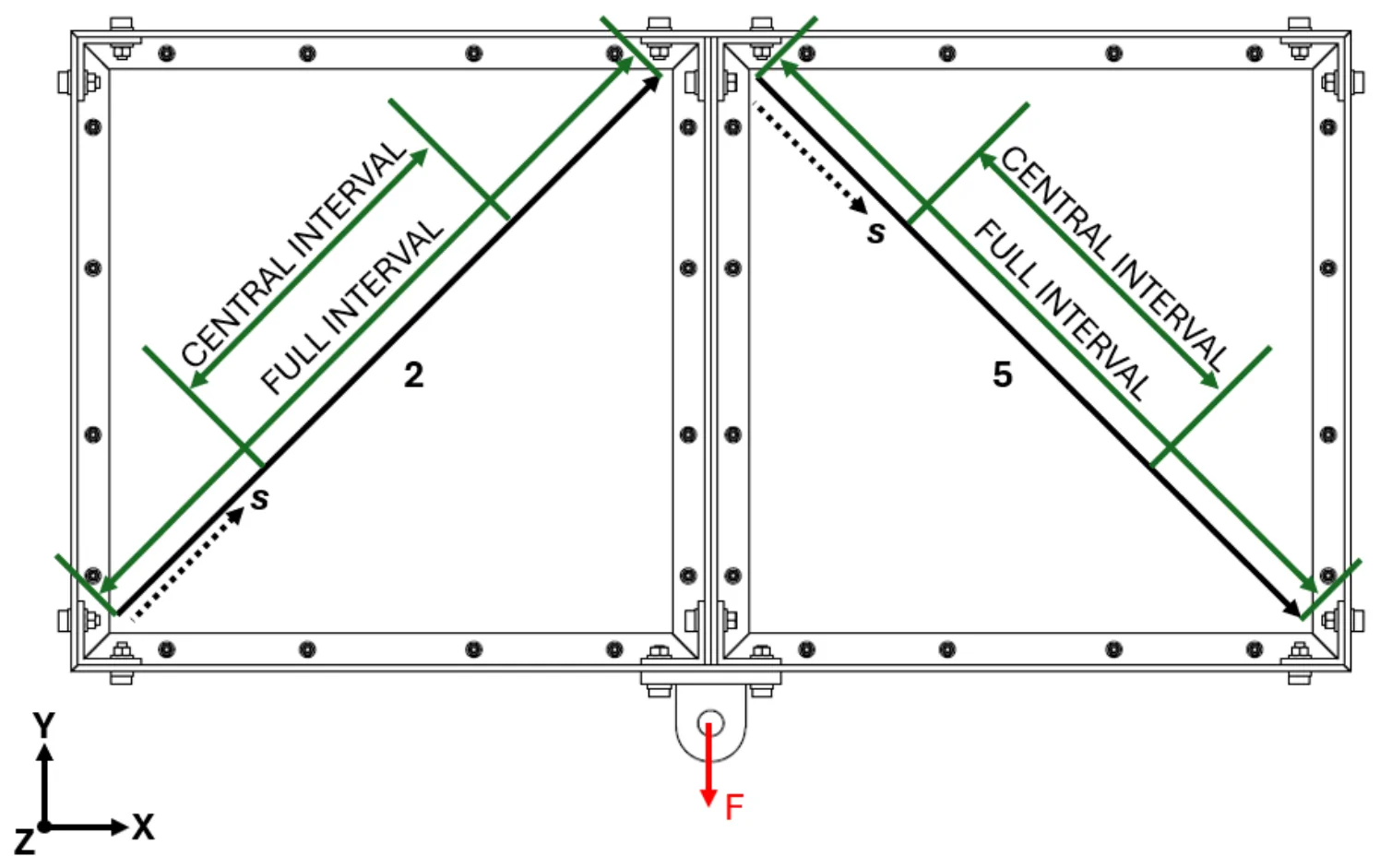
Definition of the fiber center interval along fiber paths 2 and 5.

**Figure 20:**
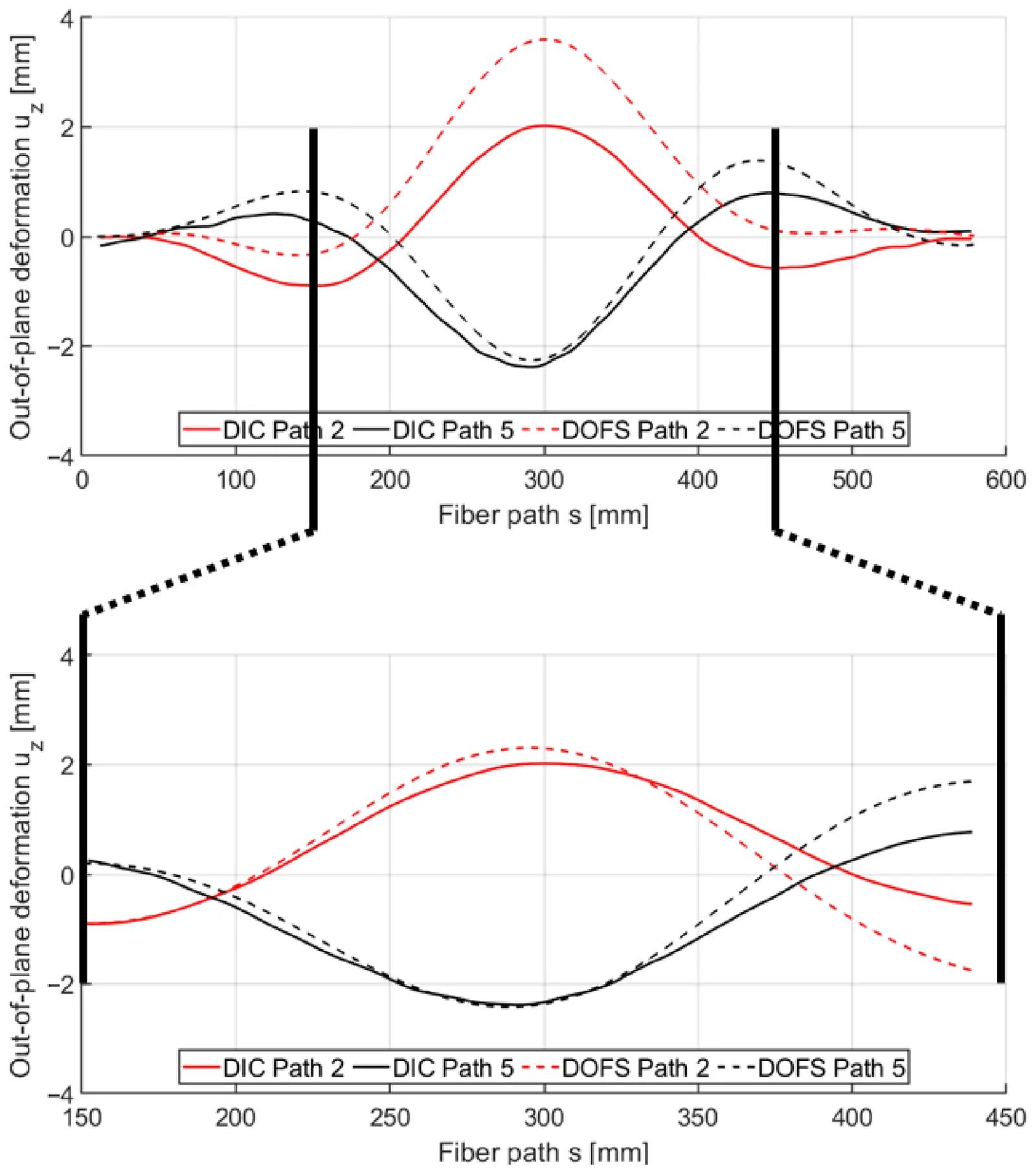
Out-of-plane displacement $u_{z}$ comparison between DOFS and DIC at 10 kN applied force using two different intervals for the calculation based on DOFS strain measurements.

In addition to imperfections, there is also the influence of mismatch arising from out-of-plane displacement predictions based on Ko’s displacement theory. The theory was developed for a beam under bending loading; however, in this investigation, the theory is used to predict the behavior of a fully constrained shear-loaded panel. The boundary condition effects in the present case differ from those in the original beam case for which the theory was developed. Restricting the reconstruction to the central portion of the fiber path is intended to reduce the influence of localized disturbances near the fixtures. The investigation was conducted by comparing the out-of-plane displacement $u_{z}$ calculated by the DOFS method along the whole length of fiber paths 2 and 5 or along a center interval within fiber paths 2 and 5 in Figure 19 and Figure 20. Center interval 1 was selected between s=150 mm and s=450 mm along fiber paths 2 and 5, meaning that every point lies at least 100 mm from the inner edge of the frame. This distance corresponds to at least ten times the 10 mm width of the effectively clamped strip and provides a geometric rationale for reducing the influence of localized clamping disturbances on the reconstruction.

The comparison shows significantly improved agreement between DIC and DOFS within the center interval, particularly for the primary diagonal wave. By only considering the center interval, the amplitude deviations between the DIC and DOFS methods decrease to 10% for path 2 and 0% for path 5 regarding the primary diagonal wave. This is a significant improvement regarding the out-of-plane calculation compared to the full interval; however, there are still increased deviations in the vicinity of the secondary diagonal wave peak at s=400–450 mm along the fiber paths. This deviation raises questions about whether the boundary conditions and their resulting membrane strains are the primary cause of the deviations. Further investigations should focus on this subject by examining the impact on bending beams or compressed plates in order to understand the fundamental effects of boundary conditions on Ko’s displacement theory. The observed sensitivity to the reconstruction domain highlights the importance of the strain field near the fixtures. Localized membrane strain contributions and complex curvature distributions may affect the application of the beam-based reconstruction to the constrained shear-loaded plate. Restricting the integration to the central interval reduces the contribution of these near-fixture regions and improves agreement with DIC for the primary diagonal wave. This provides experimental support for the selected reconstruction domain in the investigated configuration. A detailed characterization of the strain and curvature distributions near the fixtures would further support the extension of this approach to other geometries and boundary conditions.

To assess the sensitivity of the reconstruction to the selected integration domain, two additional intervals were considered alongside center interval 1 (s=150–450 mm). Center interval 2 extends from s=100 mm to s=500 mm, while center interval 3 extends from s=50 mm to s=550 mm. The corresponding peak out-of-plane displacements for fiber paths 2 and 5 at an applied load of 10 kN are reported in Table 3.

Table 3 shows that the peak displacement magnitudes obtained using center intervals 1 (s=150–450 mm) and 2 (s=100–500 mm) remain relatively close, whereas extending the integration domain to center interval 3 (s=50–550 mm) produces substantially larger values for both paths. This sensitivity to the inclusion of regions closer to the fixtures supports the choice of restricting the reconstruction to a central interval in order to reduce boundary-related effects. Together with the comparison against DIC, these results support the selected interval of s=150–450 mm for the investigated configuration.

**Figure 21:**
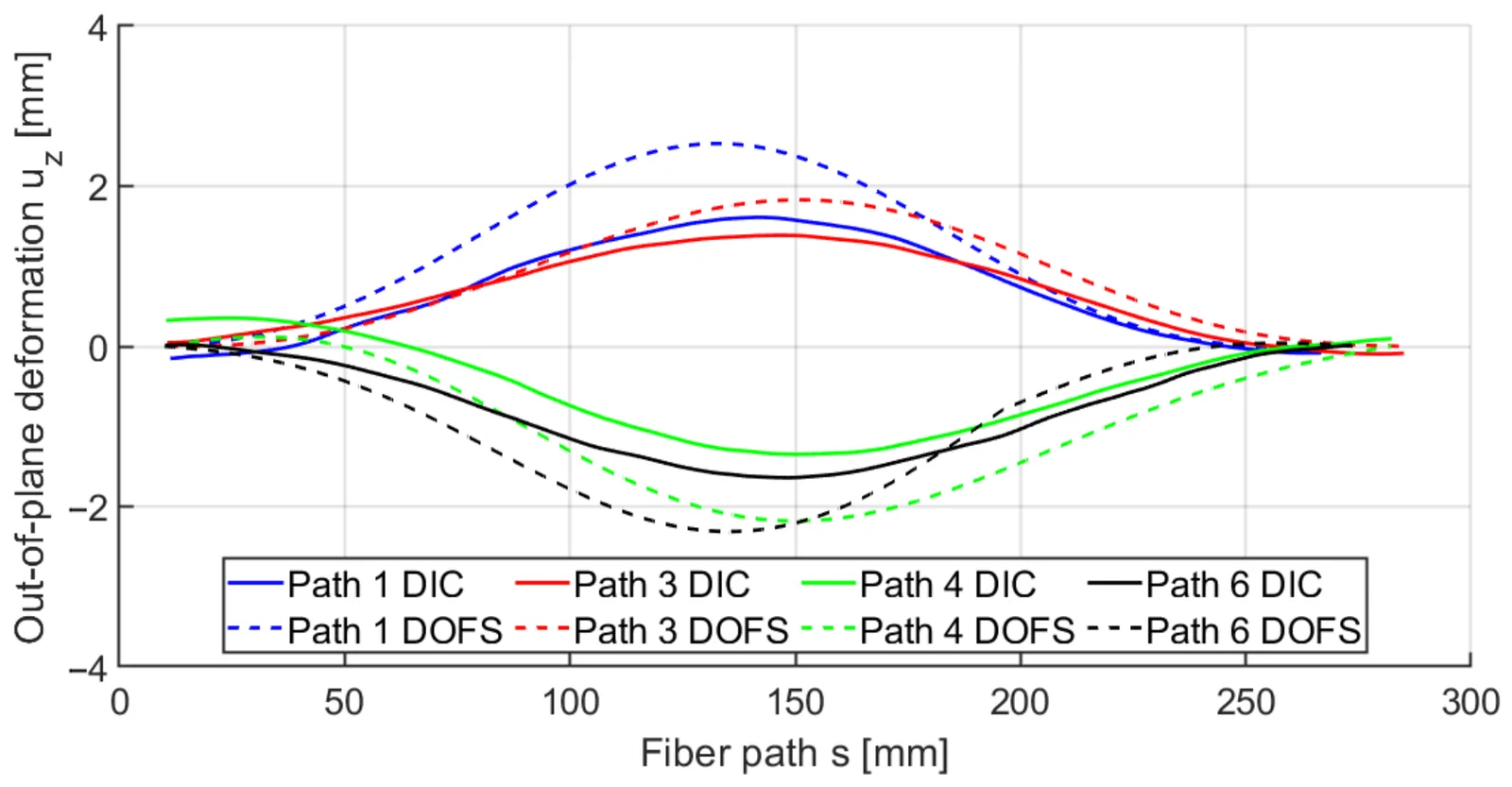
Comparison of out-of-plane displacement $u_{z}$ estimations by DIC and DOFS for fiber paths 1, 3, 4, and 6 at 10 kN applied force for Trial 1.

Figure 21 presents the out-of-plane displacement $u_{z}$ comparison for fiber paths 1, 3, 4, and 6 at an applied force of 10 kN.

For these paths, the pure bending strain cannot be calculated, as no back-to-back SGs were installed in the vicinity of paths 1, 3, 4, and 6. Consequently, the out-of-plane displacement distribution calculated by Ko’s displacement theory is based on the total measured strain.

Both the DIC and DOFS methods predict the same primary diagonal wave. The DOFS-based measurement method consistently predicts larger displacement amplitudes than the DIC measurements, with deviations of approximately 30 to 40%. In addition, differences are observed in the predicted peak locations along the fiber paths.

The lower deviation of the in- and out-of-plane displacement $u_{z}$ along fiber paths 1, 3, 4, and 6 compared to fiber path 2 raises the question of whether boundary conditions strongly influence the predictive capabilities of Ko’s displacement theory based on DOFS strain measurements. Normally, one would expect that because membrane strains near the boundary have a greater impact, the difference would be more significant. One explanation could be that in addition to membrane strains, we lack a clear post-buckled shear plate with a known post-buckling shear wave and tension diagonal; therefore, strain effects from other stresses could misrepresent the calculated out-of-plane displacements $u_{z}$.

Based on the current limitations of applying Ko’s displacement theory to DOFS strain measurements, further developments are required in order to incorporate boundary effects into the theory and enable full-field out-of-plane displacement estimation. Additionally, the manufacturing imperfections associated with the bonding process must be reduced by ensuring a uniform adhesive layer for the fiber. This limitation can be mitigated by embedding DOFS directly within composite laminates, where the fiber’s position and orientation are inherently controlled. In this preliminary proof-of-concept configuration, the DOFS system becomes a realistic and highly promising option for future aircraft structures due to its negligible weight and compactness, offering a practical alternative to DIC, which remains suitable for laboratory testing but is not feasible for operational use because of its installation requirements.

Several key operational challenges must be overcome before in-flight structural health monitoring can be realized using DOFS. First, full-scale aerospace applications expose sensing systems to complex multi-axial dynamic loading and mechanical vibrations. High-frequency structural vibrations require sampling rates significantly higher than those utilized in laboratory quasi-static testing as well as robust optical fiber bonding procedures that can prevent fatigue-induced strain transfer degradation. Second, because ambient flight conditions introduce severe transient thermal gradients, uncompensated thermal expansion could obscure the shear strain signatures in the DOFS system; thus, future field-oriented implementations must integrate temperature compensation mechanisms such as decoupled reference fibers, hybrid FBG-DOFS configurations, or multi-wavelength interrogation techniques.

## 5. Conclusions

This work compares two structural out-of-plane displacement estimation techniques: DIC was used to directly measure the out-of-plane displacement, while DOFS was used to measure strain and compute out-of-plane displacement based on Ko’s displacement theory using bending strain measurements. The comparison was made for a Wagner beam with two shear-loaded aluminum alloy panels. The comparison between the out-of-plane displacements measured by DIC and those reconstructed from DOFS strain data using Ko’s displacement theory showed good agreement for the primary diagonal wave within the selected central interval, where boundary-related effects are reduced; however, it is still necessary to extend and refine Ko’s formulation further in order to account for boundary effects. For improved accuracy, it would be convenient to develop a more specific bonding technique that minimizes imperfections introduced during the bonding process. This result represents the first step toward improving future practical health and structural displacement monitoring systems for application to lightweight and flexible aerospace structures.

## Figures and Tables

**Table 1:** Comparison of displacement $u_{z}$ and strain ε measured by DIC, strain gauges, and dial indicators, including the measurement uncertainty.

Trial	Point	Displacement $u_{z}$ (mm)			Strain ε (με)		
		DIC	Dial	Error (%)	DIC	SG	Error (%)
1	A2	2.0±0.03	2.1±0.05	4.7	262±9	280±0.7	−6.8
	A5	−2.4±0.02	−2.3±0.05	−4.3	−481±5	−515±0.9	−7.1
2	A2	2.0±0.04	2.1±0.05	4.7	245±9	280±0.5	−14.2
	A5	−2.3±0.03	−2.3±0.05	0.0	−493±6	−520±0.7	−5.5
3	A2	2.0±0.03	2.1±0.05	4.7	235±8	286±0.9	−21.7
	A5	−2.4±0.03	−2.3±0.05	-4.3	−487±5	−528±1.2	−8.4
4	A2	2.1±0.04	2.2±0.05	4.5	280±8	298±1.1	−6.4
	A5	−2.4±0.03	−2.4±0.05	0.0	−550±3	−550±0.8	0.0

**Table 2:** Comparison of strains measured by DOFS and SG at the center positions of the plates, including DOFS strains corrected to the SG sensing distance from the plate mid-plane, relative errors, and corresponding out-of-plane displacements $u_{z}$ reconstructed from DOFS measurements.

Trial	Point	Strain ε (με)				Displacement *u_z_* (mm)
		DOFS	SG	*DOFS_h_SG__*	Error (%)	
1	A2	−674±15	−551±0.6	−535±29	−3.0	2.102±0.003
	A5	270±9	235±0.8	245±17	4.1	−2.203±0.002
2	A2	−700±12	−555±0.5	−558±19	0.5	2.004±0.002
	A5	275±8	233±0.4	224±14	−4.0	−2.015±0.002
3	A2	−703±14	−562±0.6	−562±25	0.0	2.023±0.003
	A5	289±9	237±0.4	241±16	1.7	−2.027±0.002
4	A2	−717±22	−582±0.8	−570±33	−2.1	2.043±0.004
	A5	295±10	249±0.4	245±18	−1.6	−2.213±0.002

**Table 3:** Signed peak out-of-plane displacements $u_{z}$ (mm) reconstructed from DOFS measurements over three integration intervals at an applied load of 10kN.

Fiber Path	Center Interval 1	Center Interval 2	Center Interval 3
Path 2	2.1	2.2	3.8
Path 5	−2.2	−2.5	−3.7

## Data Availability

The original data presented in the study are openly available in Zenodo at https://doi.org/10.5281/zenodo.18600737.

## Abbreviations

The following abbreviations are used in this manuscript:

DIC	Digital Image Correlation
DOFS	Distributed Optical Fiber Sensor
HUMS	Health and Usage Monitoring System
LVDT	Linear Variable Differential Transformer
OF	Optical Fiber
SG	Strain Gauge
